# Discovery of CTCF-Sensitive Cis-Spliced Fusion RNAs between Adjacent Genes in Human Prostate Cells

**DOI:** 10.1371/journal.pgen.1005001

**Published:** 2015-02-06

**Authors:** Fujun Qin, Zhenguo Song, Mihaela Babiceanu, Yansu Song, Loryn Facemire, Ritambhara Singh, Mazhar Adli, Hui Li

**Affiliations:** 1 Department of Pathology, School of Medicine, University of Virginia, Charlottesville, Virginia, United States of America; 2 Pharmacy Department of Affiliated Cancer Hospital of Zhengzhou University, Zhengzhou, Henan, China; 3 Department of Biochemistry and Molecular Genetics, School of Medicine, University of Virginia, Charlottesville, Virginia, United States of America; Stanford Medical School, UNITED STATES

## Abstract

Genes or their encoded products are not expected to mingle with each other unless in some disease situations. In cancer, a frequent mechanism that can produce gene fusions is chromosomal rearrangement. However, recent discoveries of RNA trans-splicing and cis-splicing between adjacent genes (cis-SAGe) support for other mechanisms in generating fusion RNAs. In our transcriptome analyses of 28 prostate normal and cancer samples, 30% fusion RNAs on average are the transcripts that contain exons belonging to same-strand neighboring genes. These fusion RNAs may be the products of cis-SAGe, which was previously thought to be rare. To validate this finding and to better understand the phenomenon, we used LNCaP, a prostate cell line as a model, and identified 16 additional cis-SAGe events by silencing transcription factor CTCF and paired-end RNA sequencing. About half of the fusions are expressed at a significant level compared to their parental genes. Silencing one of the in-frame fusions resulted in reduced cell motility. Most out-of-frame fusions are likely to function as non-coding RNAs. The majority of the 16 fusions are also detected in other prostate cell lines, as well as in the 14 clinical prostate normal and cancer pairs. By studying the features associated with these fusions, we developed a set of rules: 1) the parental genes are same-strand-neighboring genes; 2) the distance between the genes is within 30kb; 3) the 5′ genes are actively transcribing; and 4) the chimeras tend to have the second-to-last exon in the 5′ genes joined to the second exon in the 3′ genes. We then randomly selected 20 neighboring genes in the genome, and detected four fusion events using these rules in prostate cancer and non-cancerous cells. These results suggest that splicing between neighboring gene transcripts is a rather frequent phenomenon, and it is not a feature unique to cancer cells.

## Author Summary

Genes are considered the units of hereditary information; thus, neither genes nor their encoded products are expected to mingle with each other unless in some disease situations. However, the genes are not alone in the genome. Genes have neighbors, some close, some far. With RNA-seq, many fusion RNAs involving neighboring genes are being identified. However, little is done to validate and characterize the fusion RNAs. Using one prostate cell line and a discovery pipeline for cis-splicing between adjacent genes (cis-SAGe), we found 16 new such events. We then developed a set of rules based on the characteristics of these fusion RNAs, and applied them to 20 random neighboring gene pairs. Four turned out to be true. The majority of the fusions are found in cancer cells, as well as in non-cancer cells. These results suggest that the genes are “leaky”, and the fusions are not limited to cancer cells.

## Introduction

The traditional thinking is that gene fusions and their fusion products are caused by chromosomal rearrangement at the DNA level. However, a few isolated reports of RNA trans-splicing provide support for additional mechanisms for fusion RNA production in humans [1–4]. In prostate cancer, the *SLC45A3-ELK4* fusion has recently gained attention because of its biomarker potential [5–7]. Interestingly, the fusion RNA is neither a product of chromosomal rearrangement, nor is it generated via RNA trans-splicing. With the two parental genes located next to each other, and transcribing the same strand, *SLC45A3-ELK4* is produced by a mechanism of read-through, or cis-splicing between adjacent genes (cis-SAGe) [7,8].

Traditionally, true cis-SAGe events have been considered very rare in mammalian systems. In recent years, several studies using the EST database and RNA-sequencing approaches have identified fusion RNAs involving neighboring genes, which were named “transcription-mediated gene fusions”, “tandem chimerism” and “conjoined genes” by various groups [9,10]. However, no effort has been made to characterize these fusions in terms of their generating mechanism. Theoretically, fusions involving same-strand neighboring genes could be products of interstitial DNA deletion, RNA trans-splicing, or cis-SAGe. As a result, it is still unclear whether other examples of cis-SAGe truly exist, and how widespread the phenomenon actually is.

In this study, we first noticed a high percentage of potential cis-SAGe fusion RNAs in prostate cancer, as well as in their matching normal cases. We then used LNCaP cells as a model, and by integrating RNA sequencing data and CTCF silencing, we identified close to one hundred fusions. Bioinformatics analyses and experimental evidence support 16 of these fusions as true cis-SAGe events that are similar to *SLC45A3-ELK4*. By characterizing the 16 parental gene pairs, we developed a set of rules, which were then used to identify novel fusion RNAs between neighboring genes. Interestingly, most of these fusion RNAs are also present in non-cancerous cells, indicating that cis-SAGe is not unique to cancer cells.

## Results

### Transcriptome analyses revealed high percentages of fusion RNAs involving neighboring genes

**Fig 1:**
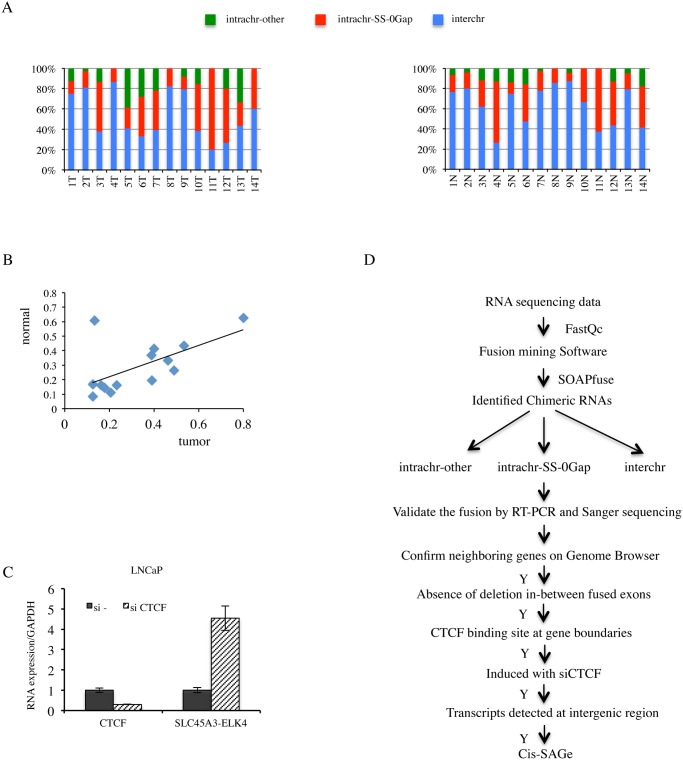
High percentage of fusion RNAs involving neighboring genes, and strategy for identification novel cis-SAGe chimeric RNAs. **A,** fusion RNAs categorized into INTRACHR-SS-0GAP, INTRACHR-OTHER and INTERCHR. Percentages of each category in individual tumor (upper) and matched normal (lower) prostate samples were plotted. **B,** correlation of the percentage of INTRACHR-SS-0GAP fusions in matched tumor and normal cases. Peason R = 0.6. **C,** CTCF knockdown induced chimeric *SLC45A3-ELK4* RNA expression. LNCaP cells were transfected with either si—or siCTCF. *CTCF* and *SLC45A3-ELK4* expression were monitored by qRT-PCR. Transcript amount was normalized to internal control GAPDH. The level of these transcripts was set to 1 in si- transfected cells. **D**, experimental flow for identification and validation of cis-SAGe events. Quality of RAW sequencing data was checked using FastQC. Paired reads were mapped to both human genome and transcriptome to identify chimeric RNAs using SOAPfuse software. Three groups of chimeric RNAs, classified by genomic features between two parental parts, were validated by RT-PCR and Sanger sequencing. Five additional steps were then applied to remove potential non-neighboring fusions, or fusions resulting from interstitial deletion, and to identify cis-SAGe events.

RNA-seq and various software tools have been used to identify fusion events in prostate cancer [5,11–13]. We used SOAPfuse [14] to analyze RNA-sequencing data [15] from 14 pairs of matched prostate normal and cancer samples. Over 300 fusion events were observed in both the normal and cancer groups. We categorized these fusion RNAs into three groups: fusions involving parental genes located on different chromosomes (INTERCHR), fusions involving neighboring genes transcribing the same strand (INTRACHR-SS-0GAP), and other fusions with parental genes on the same chromosome (INTRACHR-OTHER) (Fig. 1A). On average, around 30% of chimeric RNAs are in the category of INTRACHR-SS-0GAP (33% in cancer, 29% in normal groups), candidates for cis-SAGe. Interestingly, the normal and cancer pairs tend to have similar percentages of these fusions (ranging from 9% to 80%) (Pearson’s R = 0.6) (Fig. 1B). As these normal samples are adjacent histologically “normal” tissues from prostate cancer patients, they are not true non-neoplasia. We then analyzed RNA sequencing data from four non-cancer donors’ prostate tissues [16]. The percentage of INTRA-SS-0GAP fusions were also close to 30% (33%, 33%, 25% and 38%) (S1 Fig.). These findings suggest that cis-SAGe may be a more frequent event, and that it occurs both in normal and cancer cells. To gain a better understanding of the process, we decided to use the LNCaP prostate cancer cell line as a model.

### Transcriptome sequencing of human prostate cancer LNCaP cells

Previously, we showed an inverse correlation between the fusion RNA *SLC45A3-ELK4* expression and transcription factor CTCF binding to the insulators located at the two parental gene boundaries [7]. Consistent with a negative role in regulating cis-SAGe, silencing CTCF resulted in an induction of *SLC45A3-ELK4* fusion expression in LNCaP cells (Fig. 1C). In contrast, CTCF has been shown to facilitate the juxtaposition of interchromosomal regions [17], thus facilitating trans-splicing events. Indeed, silencing CTCF resulted in a down-regulation of a trans-spliced fusion RNA *JAZF1-JJAZ1* [7], suggesting that changes in CTCF expression can have opposite effects on a fusion RNA depending on its generating mechanism. We reasoned that more cis-SAGe fusions could be uncovered based on their response to CTCF silencing.

RNA extracted from LNCaP cells transfected with siRNA against CTCF (siCTCF), or negative control siRNA (si-) were processed, and sequenced by two different companies using the Illumina Hi-seq platform to generate two output sequences: paired-end 50-nucleotide and 101-nucleotide in read length (S1 Table.). Nearly 100 million and 50 million raw reads were yielded from each sample respectively. We used FastQC to confirm the quality of the raw fastq sequencing data, and SOAPfuse software to detect fusion transcripts. To identify cis-SAGe events, we applied the following criteria outlined in Fig. 1D: 1) fusion RNAs involving neighboring genes transcribing the same strand (INTRACHR-SS-0GAP); 2) absence of interstitial DNA deletion in-between the two fused exons; 3) presence of CTCF binding site at gene boundaries; 4) chimeric RNAs induced by siCTCF; and 5) presence of intergenic transcript.

### Classifications of the fusion RNAs

**Fig 2:**
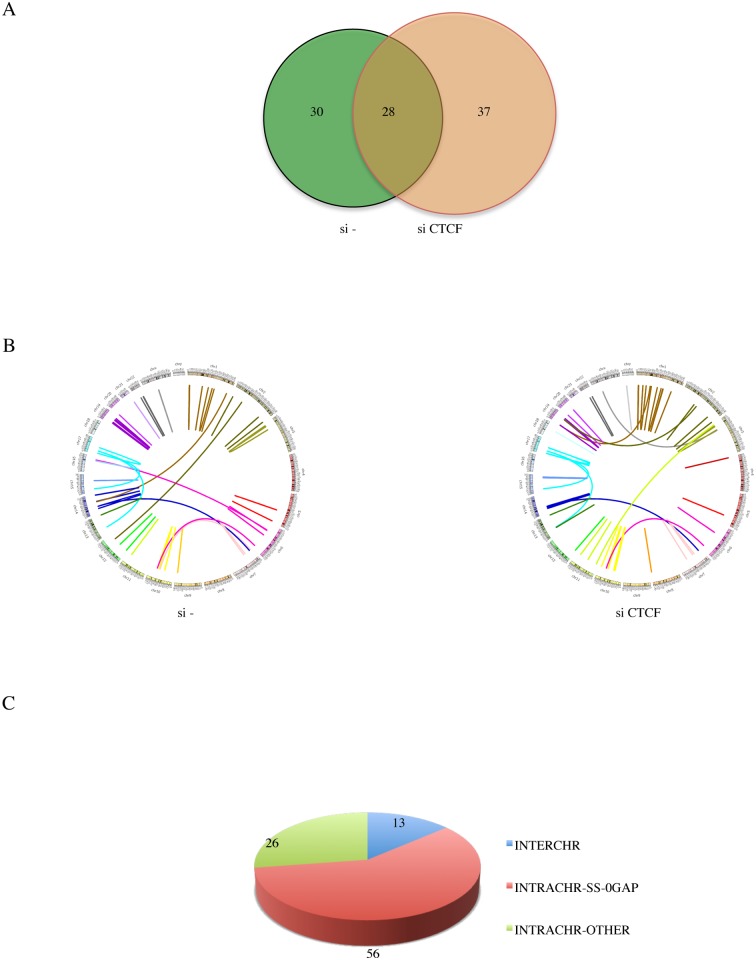
Landscape of software-identified chimeric RNAs. **A**, Venn gram showing the number of fusions in si- and siCTCF groups. **B,** Circos plot depicting chimeric RNAs discovered across the genome. Ring: chromosomes. Within the ring, lines denote the chimeric RNAs connecting two parental genes. **C**, putative chimeric RNAs were categorized into INTERCHR, INTRACHR-SS-0GAP, and INTRACHR-OTHER.

In order to identify more fusion events, we combined the reads from 50 bp read-length with those of 101 bp read-length. A total of 95 fusions (64 unique parental gene pairs) were identified from the si—and siCTCF samples (Fig. 2A, S2 Fig., and S2 Table.). Illustrated by Circos plots, it is obvious that the majority of the fusions are intrachromosomal (Fig. 2B). Out of the 95 fusions, 56 are composed of exons belonging to the INTRACHR-SS-0GAP category. There are only 13 interchromosomal fusions. The remaining 26 are fusions joining genes on the same chromosome on different strands (DS), or with more than one gene in between (INTRACHR-OTHER) (Fig. 2C). cBioPortal query on TP53 showed only 14.7% alteration in TCGA in the prostate cancer set. Consistently, LNCaP cells contain wild type TP53 [18]. This could be the reason for the relatively lower incidence of interchromosomal fusions.

We chose 71 fusions (including 48 INTRACHR-SS-0GAP, 12 INTERCHR and 11 INTRACHR-OTHER) to validate by RT-PCR. The PCR products were gel-purified, and sequenced by Sanger sequencing. A total of 62 were confirmed (one example of each category in S3 Fig., S4 Fig., and S5 Fig.). Interestingly, a higher percentage of the INTRACHR-SS-0GAP was confirmed by this method (95.8%), compared with 90.9% for INTRACHR-OTHER, and 50% for INTERCHR. We then focused on the 46 INTRACHR-SS-0GAP fusions that are composed of unique parental gene pairs as candidate cis-SAGe fusions.

### Further validation for cis-SAGe fusion RNAs

**Immediate neighboring genes.** When viewed on the hg19 Assembly of UCSC Genome Browser, we found that 8 out of the 46 candidate pairs have other gene transcripts in between (one example in S6A Fig.), raising concerns about whether or not they are truly immediate neighboring genes (Table 1). To avoid complications, we then selected the remaining 38 pairs to continue the validation.

**Table 1:** Identification of the cis-SAGe fusions.

	Fusion genes	Sanger sequencing	neighboring Gene	deletion	CTCF binding	induction by siCTCF	Intergenic transcript
1	SLC39A1-CRTC2	Y	Y	N	Y	N	
2	SMG5-PAQR6	Y	Y	N	Y	N	
3	METTL10-FAM53B	Y	Y	N	Y	N	
4	TFDP1-GRK1	Y	N	N	Y	Y	
5*	ZNF592-ALPK3	Y	Y	N	Y	Y	Y
6*	MFGE8-HAPLN3	Y	Y	N	Y	Y	Y
7	KIAA0753-PITPNM3	Y	Y	N	Y	N	
8	CIRBP-C19orf24	Y	Y	N	Y	N	
9*	PROM2-KCNIP3	Y	Y	N	Y	Y	Y
10	TP53RK-SLC13A3	N					
11	LINC00680-GUSBP4	Y	N	N	Y	Y	
12*	TMED4-DDX56	Y	Y	N	Y	Y	Y
13	PPP1R16A-GPT	Y	Y	N	Y	N	
14	TTTY15-USP9Y	Y	Y	N	Y	N	
15*	ADCK4-Numbl	Y	Y	N	Y	Y	Y
16	ADSL-SGSM3	Y	Y	N	Y	N	
17	AKAP8L-AKAP8	Y	Y	N	Y	N	
18*	AP5S1-MAVS	Y	Y	N	Y	Y	Y
19*	BAIAP2L2-SLC16A8	Y	Y	N	Y	Y	Y
20*	C14orf80-TMEM121	Y	Y	N	Y	Y	Y
21	CHCHD10-VPREB3F	Y	N	N	N	N	
22*	CLN6-CALML4	Y	Y	N	Y	Y	Y
23*	CTNNBIP1-CLSTN1	Y	Y	N	Y	Y	Y
24*	D2HGDH-GAL3ST2	Y	Y	N	Y	Y	Y
25	DMC1-DDX17	Y	Y	N	Y	N	
26	DMKN-KRTDAP	Y	Y	N	Y	N	
27	DPM2-PIP5KL1	Y	Y	N	Y	N	
28	EIF3K-ACTN4	Y	Y	N	N	N	
29	HDAC8-CITED1	Y	Y	N	Y	N	
30	MED12-NLGN3	Y	Y	N	Y	N	
31*	NUDT14-JAG2	Y	Y	N	Y	Y	Y
32*	PRIM1-NACA	Y	Y	N	Y	Y	Y
33	RRM2–C2orf48	Y	Y	N	Y	N	
34*	SCNN1A-TNFRSF1A	Y	Y	N	Y	Y	Y
35	SIDT2-TAGLN	Y	N	N	Y	N	
36	SLC29A1-HSP90AB1	Y	Y	N	Y	N	
37	TRADD-B3GNT9	Y	Y	N	Y	N	
38	WRB-SH3BGR	Y	N	N	Y	N	
39	AZGP1-GJC3	Y	Y	N	Y	N	
40	BRCA1-VAT1	Y	N				
41	DTD2-HEATR5A	Y	Y	N	N	Y	
42*	MBD1-CCDC11	Y	Y	N	Y	Y	Y
43	RNF4-FAM193A	Y	Y	N	Y	N	
44	CTBS-GNG5	Y	N	N	Y	Y	
45	DHRS1-RABGGTA	N					
46	VAMP1-CD27-AS1	Y	N	N	N	N	
47*	LMAN2-MXD3	Y	Y	N	Y	Y	Y
48	POLA2-CDC42EP2	Y	Y	N	Y	N	

The final 16 that satisfied 6 criterions are noted with *.

**Fig 3:**
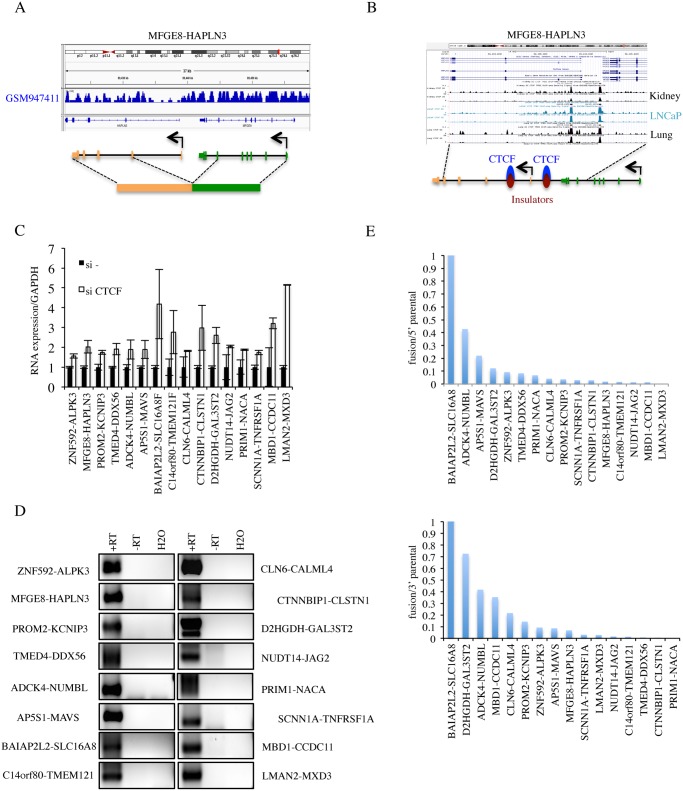
Procedures to further identify cis-SAGe fusions. **A**, cis-SAGe fusions should not have interstitial deletions between fused intergenic exons. Shown is the sequencing data of GSM947411 for the *MFGE8-HAPLN3* fusion as one example. **B**, we required the cis-SAGe fusions to contain CTCF bindings in the intergenic region between two parental genes. Shown is CTCF ChIP-seq data on kidney, LNCaP, and lung in USCS genome browser for the *MFGE8-HAPLN3* fusion as one example. **C**, the relative expression of 16 fusions by quantitative RT-PCR in si- and siCTCF treated LNCaP cells. **D**, reverse transcription using antisense primers annealing to the first exons of 3′ parental genes, and PCR of the intergenic transcripts for the 16 fusions. “+RT”, with reverse transcriptase; “-RT”, no reverse transcriptase. **E,** ratios of fusion FKPM versus parental gene FKPM were plotted following an order from the highest to the lowest.

**Absence of interstitial deletion.** As one common mechanism for generating INTRACHR-SS fusions is the deletion of the sequence in-between the fused exons, we examined the copy number variation in LNCaP cells deposited on GEO database (GSM947411). We did not find convincing evidence for interstitial deletions of the genomic DNA in-between the fused exons of any of the candidates (Fig. 3A and Table 1), supporting mechanisms other than chromosomal deletion.

**CTCF binding in between parental genes.** We used two methods to evaluate the evidence for CTCF binding in-between the 38 pairs of parental genes: 1) visual examination of CTCF binding sites generated by ENCODE on the UCSC genome browser (Fig. 3B and Table 1), and 2) searching for CTCF binding site on the Insulator Database, CTCFBSDB. For fusions *CHCHD10-VPREB3F*, *EIF3K-ACTN4*, *DTD2-HEATR5A*, and *VAMP1-CD27-AS1* (S6B Fig.), there was no evidence of CTCF binding in-between the parental genes. Among these, *CHCHD10-VPREB3F* and *VAMP1-CD27-AS1* were eliminated based on the “neighboring genes” criteria. After the above three steps of elimination, 36 fusions that have at least one CTCF binding site in-between the parental genes were left for further validation.

**Induced by siCTCF.** To evaluate the induction by CTCF silencing, we performed qRT-PCR for the remaining 36 candidates (examples shown in Fig. 3C). We found that 16 fusions could be induced by silencing CTCF, 5 down-regulated and 16 unchanged (fold change greater than or equal to 1.5) (S7 Fig. and Table 1).

**Detection of intergenic transcripts.** cis-SAGe is essentially alternative splicing between the exons of neighboring genes. Therefore, transcripts in-between the two genes that are involved in cis-SAGe should be present. To further confirm the 16 candidate fusions that are up-regulated with siCTCF, we used RT-PCR to detect intergenic transcripts. DNase treatment eliminated most, if not all, DNA contaminates in the RNA samples manifested by the absence of signal in the “no reverse transcriptase” control (examples in S8 Fig.). Intergenic transcripts were detected in all 16 candidates. To further confirm the generating mechanism, we used antisense primers of downstream parental genes for reverse transcription, and detected by PCR all 16 intergenic transcripts (Fig. 3D). These results argue against the possibility that the intergenic transcripts detected are produced by antisense transcripts. For one fusion, *CLN6-CALML4*, we could also amplify and sequence confirm the primary transcript spanning from the last exon of *CLN6* to the first exon of *CALML4* (S9 Fig.).

We hypothesized that these intergenic transcripts are more likely to be induced by siCTCF like the cis-SAGe fusions. Indeed, qRT-PCR results showed that 12 out of the 16 had obvious induction when CTCF was silenced (S10 Fig.).

**Relative expression of the fusion to parental genes.** To gain insight on the relative level of the fusion RNAs to the parental genes expression, we converted the number of junction reads of the fusion RNAs to FKPM (fragments per kilobase of transcript per million fragments sequenced). Five fusions contribute to a significant portion of the total 5′ parental gene expression (>10%). Similarly, seven fusions are above 10% of the total expression of 3′ parental genes (Fig. 3E).

### Characterization of the cis-SAGe fusions

**Fig 4:**
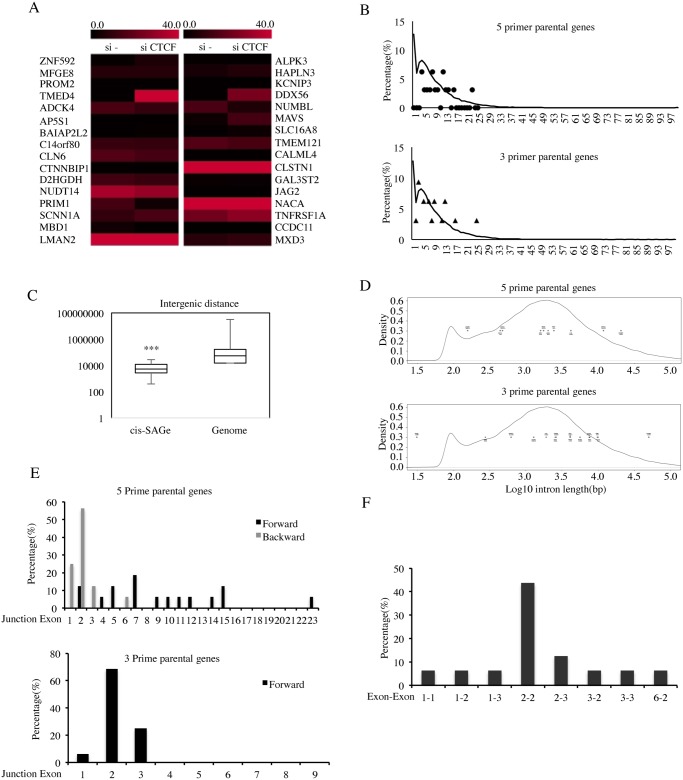
Characterization of cis-SAGe fusions. **A**, expression levels of 16-pair parental genes are plotted according to their FKPMs. **B**, the distribution of exon numbers on the 5 prime (stars) and 3 prime (triangles) parental genes versus the distribution of exon numbers for the whole genome (curve) (5prime: D = 0.7692, p-value = 2.445e-06; 3 prime: D = 0.5, p-value = 0.02123). **C**, intergenic distance of 16 cis-SAGe fusions (distance between the end of 5′ parental gene to the beginning of the 3′ gene) against the intergenic distance of the whole genome. Kolmogorov-Smirnov test D = 0.5921, p = 2.703e-05. **D**, the length of the intron after the 5 prime parental exons (upper) and the length of the intron before the 3 prime parental exons (lower) against the intron length of whole genome. No statistical significance was observed (5prime: D = 0.2086, p-value = 0.7769; 3 prime: D = 0.2996, p-value = 0.1353). **E**, the distribution of exonic position of the immediate exons to the fusion junction in the 5 prime (forward and backward) and 3 prime genes. **F**, the distribution of the most common combinations of fused exons (for 5′ gene, exon position is counted backward). ***p<0.001.

We noticed that the FKPMs of these 32 parental genes range from 0 to 122 (Fig. 4A). Considering the highest FKPM in the RNA-seq is above 2800, it is thus unlikely that the cis-SAGe fusions are non-specific side products, all due to overwhelming quantities of parental gene transcripts. The expression of several fusions contributes significant portions of parental genes (Fig. 3E), further supporting the argument. In addition, the fact that all of these 16 fusion RNAs were induced by siCTCF, yet many parental genes were not, suggests that the cis-SAGe events are actively regulated by additional mechanisms other than their parental genes’ expression.

Previous analyses reported that in the human genome, roughly 25,000 genes are composed of over 200,000 exons, with a typical gene containing 8.7 exons, and an average exon length of 174.5bp [19]. We used Genes and Gene Prediction Tracks from hg19 Assembly to download gene features, including exon number. The density of genes containing certain numbers of exons are plotted in Fig. 4B. In the genome, a high percent of genes have a single exon (12%). Another peak represents genes with about five exons (~ 8%). The overall distribution of exon numbers of the cis-SAGe parental genes is different from this whole genome analysis, especially for the 5′ parental genes (p = 2.445e-06) (S11 Fig.). Notably, none of the cis-SAGe parental genes are single-exonic.

In order to investigate whether the distance between the neighboring genes plays a role in the generation of cis-SAGe fusions, we analyzed the distribution of the distances between neighboring genes transcribing the same strand within the genome. The median distance between such neighboring genes in the genome is around 54 Kb (Fig. 4C). We found a strong statistical difference between the 16 pairs of cis-SAGe parental genes and the whole genome analyses, as the longest distance out of the 16 pairs is less than 30kb (p = 2.703e-05).

It is known that longer introns tend to facilitate alternative splicing, as RNA pol II has a higher chance of pausing on longer introns [20]. We compared the intron size involved in cis-SAGe fusions (for 5′ genes, the intron after the fused exon; for 3′ genes, the intron before the fused exon) with the hg19 genome (Fig. 4D). No statistical difference was noted (p = 0.7769, and 0.1353 for 5′ and 3′ genes respectively).

We then used CisFinder [21] to identify potential DNA motifs in the fused exons and immediate introns. No consistent motif in the introns, or in the 5′ and 3′ exons was found in the 16 pairs.

To determine whether a certain exon is favored for a cis-SAGe event, the positions of the exons immediately next to the fusion junction site, relative to the parental genes were plotted (Fig. 4E). No obvious “hot exonic position” was observed for the 5′ parental genes, but strikingly, more than 68% of fusion junctions have been observed occurring at the second exon of the 3′ parental genes (11 out of 16) (Fig. 4E). If the participation of the exons are totally random, the chances of having 11 out of the 16 fusions involving the 2nd exon of the 3′ gene is very low, illustrated by a simulation test (p = 1.718378e-05) (S12 Fig.). On the other hand, the bias towards shorter intergenic distance shown earlier does support the frequent usage of the 2nd exon, as it is the closest exon that has a splicing acceptor sequence. However, arguing against a totally non-specific model, there are three fusions that use the first exon of their 3′ parental genes (*TMED4-DDX56*, *NUDT14-JAG2*, and *PRIM1-NACA*), and no common splicing acceptor site (AG or AC), which account for over 99.98% of known splicings [22], was found in the sequence before these first exons.

Considering that the distance between the spliced exons may be one key factor, we went back to examine the distribution of 5′ exon positions counting backward. Impressively, we found that 50% of the fusions actually used the second-to-last exon of the 5′ parental genes (p<0.001)(Fig. 4E). When the top combinations of 5′ and 3′ exon usage were plotted, we noticed a strong bias towards the “2–2”, that is “second-to-last” of the 5′ gene fused to the second exon of the 3′ gene (Fig. 4F).

### Expression of the cis-SAGe fusion RNAs in prostate cell lines and clinical cases

**Fig 5:**
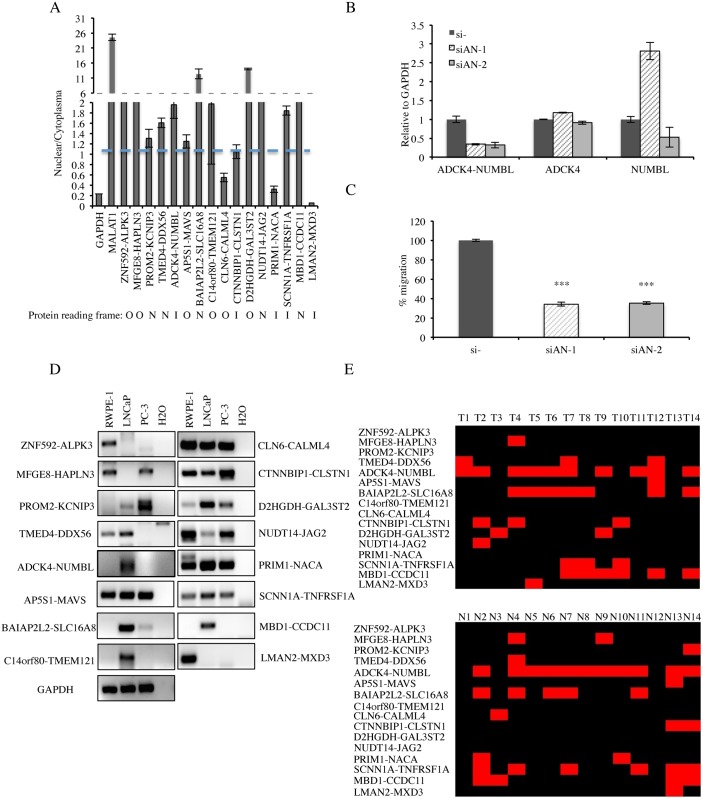
Detection of cis-SAGe chimeric mRNA in prostate cell lines and clinical tissues. **A**, distribution of 16 fusion RNAs in nuclear vs. cytoplasmic fractions. Traditional protein-coding gene *GAPDH* and a known long non-coding RNA MALAT1 were used as controls. The protein-coding potential of each fusion is marked below. N: not affecting protein coding, applies to fusions where junction sites fall in the UTR. O: out-of-frame, applies to fusions where the reading frame of the 5′ gene is different from that of the 3′ gene. I: in-frame, the reading frame of 5′ gene is the same as that of 3′ gene. **B,** qRT-PCR for *ADCK4-NUMBL* and the parental genes. LNCaP cells were transfected with siRNAs targeting the fusion RNA (si-AN1 and si-AN2). Levels of various transcripts were normalized to that in si-negative control (si-). **C,** cell motility was measured by wound healing assay. Cells were transfected with siRNAs targeting *ADCK4-NUMBL* and si-negative control. The changes of the wound size were normalized to that in the si-negative control group (n>3, p<0.0001) **D,** detection of the 16 cis-SAGe chimeras in RWPE-1 (benign prostate cell), LNCaP, and PC-3 cells by RT-PCR and followed by agarose electrophoresis. GAPDH as internal control. **E**, summary of the 16 cis-SAGe chimeric RNAs in 14 clinical prostate cancer and normal tissues. STAR software was used to align the chimeras onto the clinical RNA-seq data; samtools and IGV were used to map spanning reads across the fusion junction. Black indicates the absence of a fusion, while red indicates the detection of a fusion.

Out of the 16 fusion RNAs, five fusions have their junction sites fall into the UTR region, thus not changing protein-coding sequence (NR) (Fig. 5A). In six fusions, the protein coding sequence of the 3′ genes use a different reading frame than the 5′ gene (out-of-frame). In the remaining five, the reading frame of the 3′ gene is the same as the 5′ gene (in-frame). The number of in-frame fusions versus. that of the out-of-frame fusions (5 vs. 6) is higher than random (1 vs. 2), but the small number prevents further generalization at this stage. We then fractionated LNCaP cells, and measured the relative amount of these 16 fusion RNAs in the nuclear versus cytoplasmic fractions. Interestingly, the NR and out-of-frame fusions (10/11) were enriched in the nuclear fraction (nuclear/cytoplasma >1), indicating potential non-coding roles. In contrast, four out of five in-frame fusions showed more or equal amounts in the cytoplasmic fraction, possibly functioning as traditional protein coding mRNAs (Fig. 5A).

For one fusion RNA, *ADCK4-NUMBL*, we were able to obtain two siRNAs that specifically target the fusion transcript (Fig. 5B). They both significantly knocked down the fusion RNA, and had no obvious effect on the *ADCK4* parental transcript. si-AN2 caused a slight reduction of the *NUMBL* parental transcript, whereas si-AN1 even caused an induction of *NUMBL*. We didn’t notice any obvious change in cellular proliferation rate, but cell motility was significantly reduced when LNCaP cells were transfected with these siRNAs (Fig. 5C)

To further validate these cis-SAGe fusions in other systems, we performed RT-PCR for the final 16 fusions in LNCaP, PC3, a castration-resistant prostate cancer cell line, and RWPE-1, a non-cancer prostate epithelial cell line (Fig. 5D). The majority of the fusion RNAs can be detected in two or more lines. *ZNF592-ALPK3* and *LMAN2-MXD3* were only detectable in RWPE-1 cells, but hardly detectable in the two cancer cell lines. In contrast, three fusions, *PROM2-KCNIP3*, *BAIAP2L2-SLC16A8*, *and D2HGDH-GAL3ST2* had higher expression in the two cancer cell lines than in RWPE-1 cells.

We also performed STAR alignment [23] of the RNA-seq data from 14 clinical prostate cancer cases [15]. With IGV analyses [24], 11 out of the 16 cis-SAGe fusions were also found in this dataset (Fig. 5E). Using this method, we found 12 fusions in the matched normal group. Of note, most fusions were not cancer specific.

### Identification of novel cis-SAGe candidate RNAs

**Fig 6:**
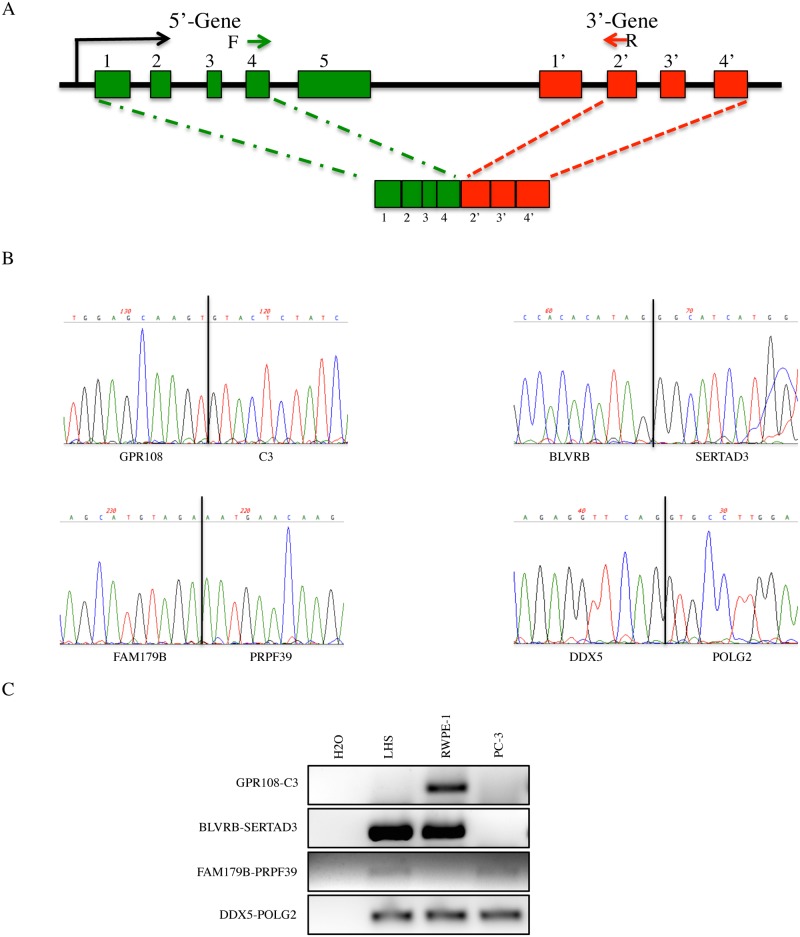
Identification of novel cis-SAGe candidate events. **A,** configuration of most common cis-SAGe events based on the 16 fusions. Bars represent exons and lines represent introns and intergenic regions. Arrows represent primers used to detect novel cis-SAGe events. **B,** Sanger sequencing of four novel fusions detected in LNCaP cells. **C,** RT-PCR of the same four fusions in LHS, RWPE-1 and PC3 cells.

Next, we wondered whether or not the rules generalized from the 16 fusions could lead us to discover novel cis-SAGe fusions. We summarized the following four rules for cis-SAGe fusions (Fig. 6A): 1) neighboring genes transcribing the same strand; 2) with an intergenic distance less than 30kb; 3) 5′ genes actively transcribing and 4) favoring a configuration of the second-to-last exon in the 5′ fused to the second exon in the 3′ parental genes. According to hg19 genome assembly, there are 9478 pairs of neighboring genes transcribing in the same strand, and that are within a 30kb distance. We then downloaded RNA-seq data for prostate samples [25], and selected 20 random pairs with the 5′ genes expressed in prostate (FPKM>1). Using primers annealing to the second-to-last exon in the 5′ and the second exon in the 3′ gene, we successfully amplified four pairs in LNCaP cells. Sanger sequencing revealed an exact exon-exon splicing pattern (Fig. 6B). All four pairs were also detected in RWPE-1, or LHS, both non-cancer prostate epithelial cell lines (Fig. 6C).

## Discussion

Traditionally, fusion RNAs containing exons of neighboring genes have been considered rare in mammalian cells, with only a handful of examples experimentally identified [26]. However, in our initial analyses of paired—end RNA-sequencing data from the 14 pairs of normal and prostate cancer cases, we found a high percentage of fusion RNAs that are candidates for cis-SAGe (averaging around 30%). This observation is consistent with other in silico analyses and sequencing efforts [9,10,27]. However, the majority of the reported fusion RNAs have not been validated, and their generating mechanisms remain unknown. In fact, some other studies have attributed many such chimeras to experimental artifacts [28], raising questions about whether these fusions are even real. In our previous study, we reported *SLC45A3-ELK4* as the first verified cis-SAGe event [7]. Here, we applied a set of criteria and identified 16 additional fusions that are generated by this cis-SAGe mechanism.

CTCF is a highly conserved zinc finger protein that binds to insulator sequences in the genome [29]. Insulators between the neighboring genes act as boundaries to protect a gene against the encroachment of adjacent, inactive, condensed chromatin, or against the activating influence of distal enhancers associated with other genes [30]. CTCF plays diverse regulatory functions, including transcriptional activation/repression, insulation, imprinting, and X chromosome inactivation [29]. Here we manipulated CTCF levels to enhance certain cis-SAGe events. To estimate that 25% of the fusions (16 out of a total number of 64 (unique parental gene pairs)) could be considered as cis-SAGe events may still be an underestimation. It is very likely that some cis-SAGe events are not regulated by CTCF. It is also possible that not all cis-SAGe events have CTCF binding in the experimental conditions we used, and/or are not induced by siCTCF. Thus, the study is aimed to discover CTCF-sensitive cis-SAGe events. Of note, this is also an artificial system created only to enhance some cis-SAGe signals in a cell line system. cis-SAGe is likely to be a complex event regulated by multiple factors. At this moment, the role of CTCF in regulating global cis-SAGe events in clinical prostate cancer is not clear.

It has been reported that CTCF could also facilitate the formation of chromosomal translocation by bringing distant genes into close proximity [31,32]. As we noted before, silencing CTCF resulted in a reduced expression of a trans-spliced chimera *JAZF1-JJAZ1* in endometrial cells [7]. The discarded fusions for this study are likely to be candidates for RNA trans-splicing or chromosomal rearrangement, especially the ones that are down-regulated with siCTCF.

Neither statically significant trends in intron size, nor consistent motifs were found in the final 16 fusions, possibly due to the small number of the these cis-SAGe events. However, the parental genes tend to be multi-exonic, and we found a strong preference for shorter intergenic distance. This may partially explain the biased involvement of the second exon in the 3′ parental gene, and the second-to-last exon in 5′ gene. Even though this configuration only applies to about half of the final 16 fusions, we were able use this rule to discover four novel fusion RNAs in 20 randomly selected neighboring genes. Interestingly, these four fusions were also found in at least one non-cancer prostate cell line.

Traditionally, fusion RNAs were thought to be uniquely expressed in cancer cells, and sought after as ideal biomarkers. Some of the fusions we found here did show differential expression between prostate cancer cells and non-cancerous cells, and are potential biomarkers. However, the finding of many new chimeras in normal clinical samples, as well as in non-cancerous cell lines suggests that these events also happen physiologically. For cis-SAGe fusions, this means that genes are more “leaky” than we previously thought.

## Materials and Methods

### Cell culture, siRNA knockdown and transfection

Prostate cancer cell lines LNCaP (androgen-dependent) were grown in RPMI1640 (Hyclone) media supplemented with 10% FBS and 1% 100 x Pen/Strep (Hyclone). siRNA against luciferase gene was used as control siRNA [33]. CTCF siRNA was purchased from Invitrogen and as described before [7]. The two siRNAs against ADCK4-NUMBL targeting sequences were TCCGCCCTTGGTTTCAAAG, and GGGUCCGCCCTTGGTTTCA. siRNA transfection was carried out using Lipofectamine RNAiMAX (Life Technologies) following the manufacturer’s protocols. Cellular fractionation was carried out according to manufactures’ protocol (NE-PER Nuclear and Cytoplasmic Extraction Kit, Thermo).

### Wound healing assay

LNCaP cells transfected with si-negative control, or siRNAs against ADCK4-NUMBL were cultured for 3 days to obtain 80–90% monolayer confluency. A wound was created by scraping the cells using a 10ul plastic pipette tip, and the medium was replaced with fresh medium. Images were captured immediately after the scratch and six hours later. Cell migration was qualitatively assessed by the size of the wounds at the end of the experiment.

### RNA extraction and sequencing

Cells transfected with si- or siCTCF were harvested 3 days after transfection. The RNA was extracted with TRIzol reagent (Life Technologies) following the manufacturer’s instruction. To assure the high quality RNA for next generation sequencing, RNA was further cleaned using the RNeasy kit (Qiagen). The mRNA in total RNA was converted into a library of template molecules suitable for subsequent cluster generation using the reagents provided in the Illumina TruSeq TM RNA Sample Preparation Kit. Millions of unique clusters on flow cells were loaded into the Hiseq 2000 platform and processed for RNA sequencing.

### Data analysis for RNA-seq data

The two samples, the negative control and siCTCF samples were sequenced by the Illumina Hi-seq platform with paired ends to reach 100 million reads with 50bp read lengths (HudsonAlpha Insitute, Huntsville, AL), or 50 million reads with 101bp read lengths (Axeq, Seoul, Korea). To check the quality of the raw data, the software FastQC was used. The deep sequencing data was mapped to Human genome version hg19, and analyzed using the SOAPfuse software [14]. The identified chimeric RNAs were presented using Circos as previously described [34]. STAR align was used to identify the final 16 fusions in the clinical prostate cancer samples downloaded from the European Bioinformatics Institute.

### RT-PCR and Sanger sequencing

Fusion candidates from SOAPfuse analyzed RNA-seq data were validated at the RNA level by real-time PCR. Quantitative RT-PCR was performed using the ABI Step One Plus real time PCR system (Applied Biosystems) following the manufactures’ instructions. We designed specific primer pairs (S3 Fig.) for the fusion candidates, intergenic transcripts and randomly selected 20 pairs, with each primer targeting one parental gene. Following RT-PCR and gel electrophoresis, all purified bands were sent for Sanger sequencing.

### Visualization

Multi Experiment Viewer (Mev, 4.9 version) software suite was used to generate the heat map containing the visualization of the expression levels of parental genes that are involved in the 16 fusions [35]. IGV analysis was used to visualize fusions in the clinical samples as described before [24].

### Statistical analyses

For intergenic distance, exon numbers, and intron size, the Kolmogorov-Smirnov test [36] was used to decide if the distributions of the 16 pair parental genes were different from those of the whole human genome, by calculating the maximum distance between the sample and population empirical/cumulative distribution function (cdf). In each case, two hypotheses were tested, H0: the distribution of the 32 parental genes follows a normal human genome distribution versus H1: it does not follow the specified distribution.

To test if the total number of fusions use exon2 of the 3′ parental genes is statistically significant, we ran a simulation, in which 10,000 random samples of fusions for the 16 3′ parental genes’ exons were created. We then counted the total number of exon2 fusions in each simulation and plotted the results, creating an approximate binomial distribution of sample size n = 16. We then calculated the probability underneath the binomial distribution in which there are 11 or more exon2 fusions, Pr (number of exon2> = 11), and found the probability to be 1.718378e-05. Thus concluding that getting 11 exon2 fusions is significant.

### Data access

The Raw and processed RNA-sequencing data from this study have been submitted to the NCBI Gene Expression Omnibus (GEO; http://www.ncbi.nlm.nih.gov/geo/) under accession number GSE63487.

The prostate clinical datasets from 14 patients were downloaded from EBI (European Bioinformatics Institute) (http://www.ebi.ac.uk/arrayexpress/experiments/E-MTAB-567) [15].

## Supporting Information

S1 Fig Percentages of each category fusions in four normal prostate samples were plotted. (PDF)

S2 Fig Landscape of fusion RNAs detected by RNA-seq and SOAPfuse in si—and siCTCF. Majority of the fusions are intrachromosomal.
(PDF)

S3 Fig *MFGE8-HAPLN3* (one of the INTRA-SS-0GAP). **A,** structure of the fusion. **B**, validation of the fusion gene breakpoint using Sanger sequencing of RT-PCR product.
(PDF)

S4 Fig *TIMM23B-LINC00843* (one of the INTRA-SS-OTHER). **A,** structure of the fusion. **B**, validation of the fusion gene breakpoint using Sanger sequencing of RT-PCR product.
(PDF)

S5 Fig *MLK4-FUT8* (one of the INTER-CHR). **A,** structure of the fusion. **B**, validation of the fusion gene breakpoint using Sanger sequencing of RT-PCR product.
(PDF)

S6 Fig Examples of eliminated fusions. **A,** *TFDP1* and *GRK1* are not immediate neighboring genes, as there is another gene, *ATF4B*, between the two. **B,** no obvious CTCF binding in-between *VAMP1* and *CD27-AS1*.
(PDF)

S7 Fig Expression level changes of the fusions determined by qRT-PCR analysis in siCTCF relative to si-. (PDF)

S8 Fig RT-PCR detected the intergenic transcripts between pairs of parental genes. Shown are 9 examples.
(PDF)

S9 Fig For *CLN6-CALML4*, primary transcript spanning the last exon of CLN6 and the first exon of CALML4 were amplified and sequenced. Red oligo is the RT primer. F and R are the primer pairs for the long-range PCR. Inter-F and Inter-R are the primer pairs for data presented in Fig. 3D.
(PDF)

S10 Fig The intergenic transcript was induced by siCTCF in 12 cases out of 16 fusions. (PDF)

S11 Fig The distribution of exon numbers on the 5 prime and 3 prime parental genes of the 16 cis-SAGe fusions versus distribution of exon numbers for all the genes in the whole genome. (5 prime: D = 0.7692, p-value = 2.445e-06; 3 prime:D = 0.5, p-value = 0.02123)
(PDF)

S12 Fig Simulation test for exon 2 usage on the 3′ parental genes. 10,000 random sets of events with variation of the exon usage of the 16 3′ genes exons were simulated. The total number of fusions using exon2 in each set was counted and the results plotted. The peak at 3 indicates that out of 16 genes, the situation that having 3 genes using exon2 is more likely than any other number of genes using exon2. The fact that we have 11 genes using exon2 (falls outside the chart) is highly significant statistically (p = 1.718378e-05).
(PDF)

S1 Table Summary of data from RNA-seq. (PDF)

S2 Table Full list of fusions in si- and siCTCF identified by RNA-seq and SOAPfuse analysis. (PDF)

S3 Table List of PCR and real-time PCR primers. (PDF)
