# Correction: Discovery of CTCF-Sensitive Cis-Spliced Fusion RNAs between Adjacent Genes in Human Prostate Cells

**DOI:** 10.1371/journal.pgen.1005161

**Published:** 2015-04-10

**Authors:** Fujun Qin, Zhenguo Song, Mihaela Babiceanu, Yansu Song, Loryn Facemire, Ritambhara Singh, Mazhar Adli, Hui Li

The term FKPM (fragments per kilobase of transcript per million fragments sequenced) is incorrect throughout. The correct description should be FPKM (Fragments Per Kilobase Of Exon Per Million Fragments Mapped).

The following references are omitted in the article:

Two references are omitted from the second sentence of the second paragraph in the Introduction. The sentence should read: In recent years, several studies using the EST database and RNA-sequencing approaches have identified fusion RNAs involving neighboring genes, which were named “transcription-mediated gene fusions”, “tandem chimerism” and “conjoined genes” by various groups [9,10], (Prakash et al., 2010), (Parra et al., 2006).

The references are:

Prakash T, Sharma VK, Adati N, Ozawa R, Kumar N, Nishida Y, et al. (2010) Expression of conjoined genes: another mechanism for gene regulation in eukaryotes. PloS one 5: e13284.

Parra G, Reymond A, Dabbouseh N, Dermitzakis ET, Castelo R,, Thomson TM, et al. (2006) Tandem chimerism as a means to increase protein complexity in the human genome. Genome research 16: 37–44.

Two references are omitted from the seventh sentence of the first paragraph under the subheading “Classifications of the fusion RNAs” in the Results section. The sentence should read: cBioPortal query on TP53 showed only 14.7% alteration in TCGA in the prostate cancer set. Consistently, LNCaP cells contain wild type TP53 [18], (Cerami et al., 2012), (Gao et al., 2013).

The references are:

Cerami E, Gao J, Dogrusoz U, Gross EB, Sumer SO, Aksoy BA, et al. (2012) The cBio Cancer Genomics Portal: An Open Platform for Exploring Multidimensional Cancer Genomics Data. Cancer Discovery. 2; 401.

Gao J1, Aksoy BA, Dogrusoz U, Dresdner G, Gross B, Sumer SO, et al. (2013) Integrative analysis of complex cancer genomics and clinical profiles using the cBioPortal. Sci. Signal. 6, pl1.

Two references are omitted from the first sentence of the first paragraph under the subheading “CTCF binding in between parental genes” under the subheading “Further validation for cis-SAGe fusion RNAs in the Results section. The sentence should read: “We used two methods to evaluate the evidence for CTCF binding in-between the 38 pairs of parental genes: 1) visual examination of CTCF binding sites generated by ENCODE on the UCSC genome browser (Fig. 3B and Table 1), and 2) searching for CTCF binding site on the Insulator Database, CTCFBSDB (Ziebarth et al., 2013), (Bao et al., 2008).

The references are:

Ziebarth JD, Bhattacharya A, Cui Y (2013) CTCFBSDB 2.0: a database for CTCF-binding sites and genome organization. Nucleic Acids Research. 41(D1): D188-D194.

Bao L, Zhou M and Cui Y (2008) CTCFBSDB: a CTCF binding site database for characterization of vertebrate genomic insulator. Nucleic Acids Research. 36: D83-D87.

Two references are omitted from the second sentence of the fourth paragraph under the subheading “Expression of the cis-SAGe fusion RNAs in prostate cell lines and clinical cases” in the Results section. The sentence should read: With IGV analyses [24], (Robinson et al., 2011), (Thorvaldsdóttir et al.,), 11 out of the 16 cis-SAGe fusions were also found in this dataset (Fig. 5E).

The references are:

Robinson JT, Thorvaldsdóttir H, Winckler W, Guttman M, Lander ES, Getz G, et al. (2011) Integrative Genomics Viewer. Nature Biotechnology 29, 24–26

Thorvaldsdóttir H, Robinson JT, Mesirov JP. (2013) Integrative Genomics Viewer (IGV): high-performance genomics data visualization and exploration. Briefings in Bioinformatics 14, 178–192.

## References

[1] Qin F, Song Z, Babiceanu M, Song Y, Facemire L, Singh R, et al (2015) Discovery of CTCF-Sensitive Cis-Spliced Fusion RNAs between Adjacent Genes in Human Prostate Cells. PLoS Genet 11(2): e1005001 doi: 10.1371/journal.pgen.1005001 25658338 10.1371/journal.pgen.1005001 PMC4450057

