# Supplementary figures and images for: Discovery of CTCF-Sensitive Cis-Spliced Fusion RNAs between Adjacent Genes in Human Prostate Cells
Source: PLoS Genet. 2015 Feb 6;11(2):e1005001. doi: 10.1371/journal.pgen.1005001 (PMC4450057; doi:10.1371/journal.pgen.1005001)

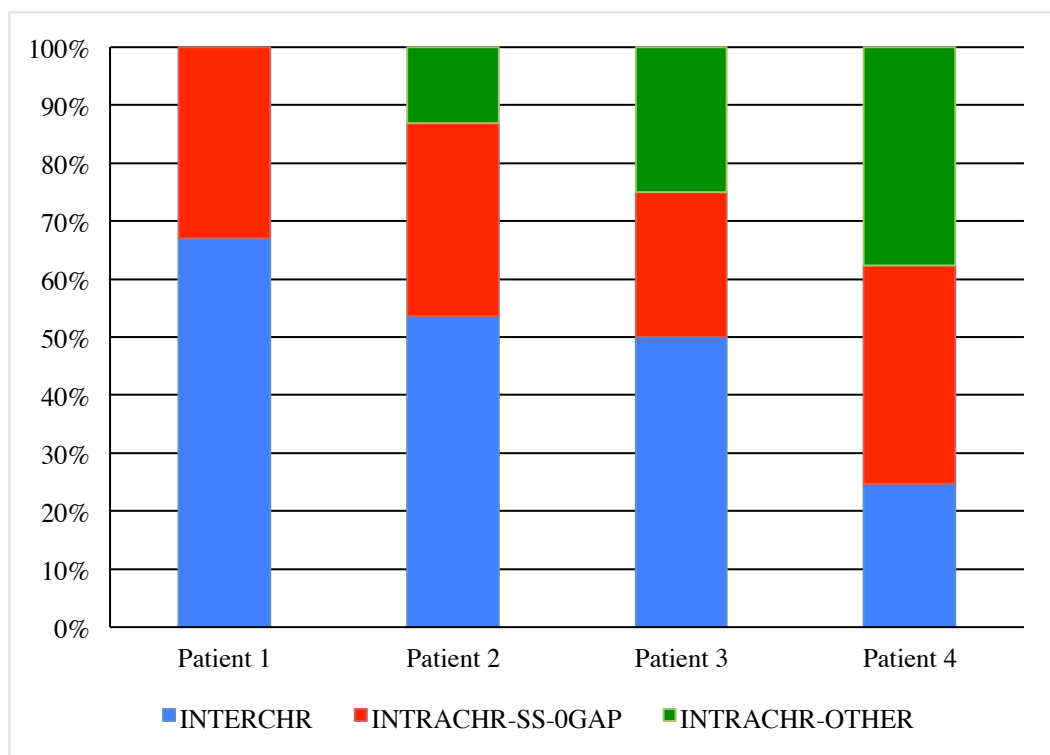

Fig. S1

Supplement: S1 Fig — (PDF) [file pgen.1005001.s001.pdf]

A

## MFGE8-HAPLN3

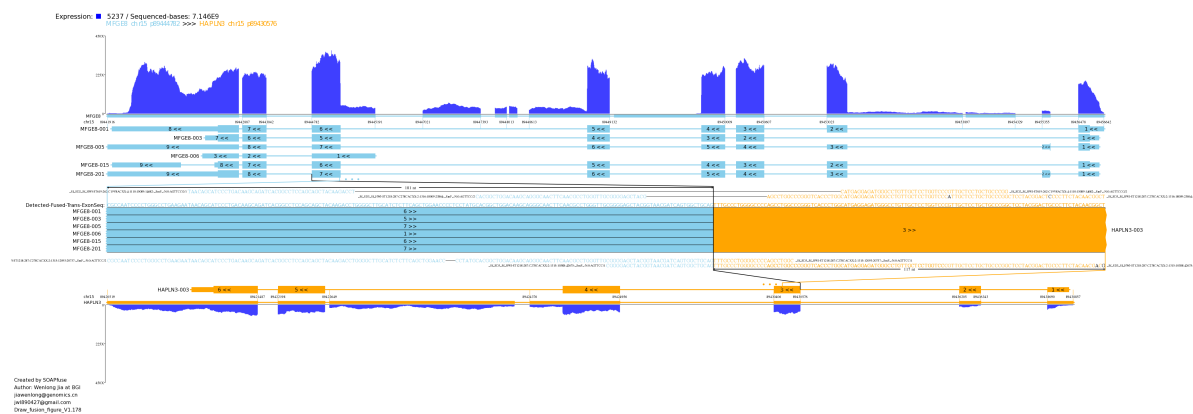

B

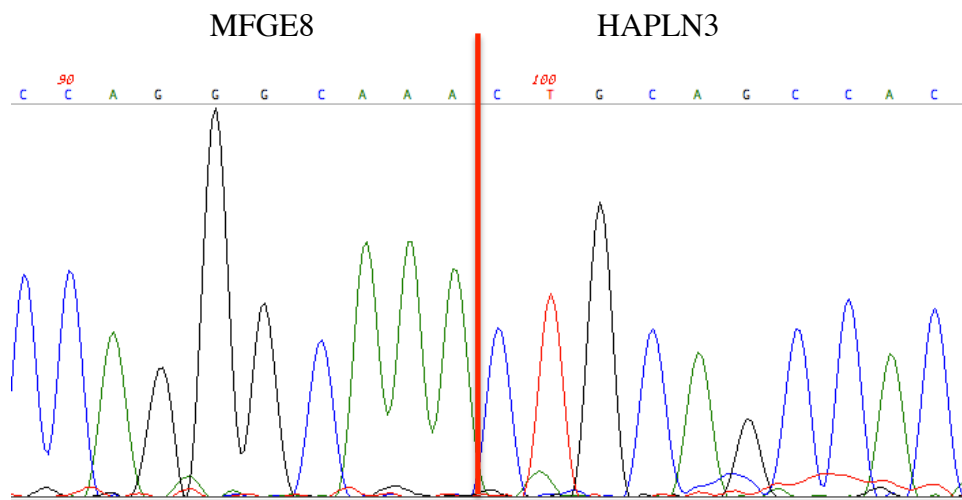

Fig. S3

Supplement: S3 Fig — A, structure of the fusion. B, validation of the fusion gene breakpoint using Sanger sequencing of RT-PCR product. (PDF) [file pgen.1005001.s003.pdf]

A

## TIMM23B-LINC00843

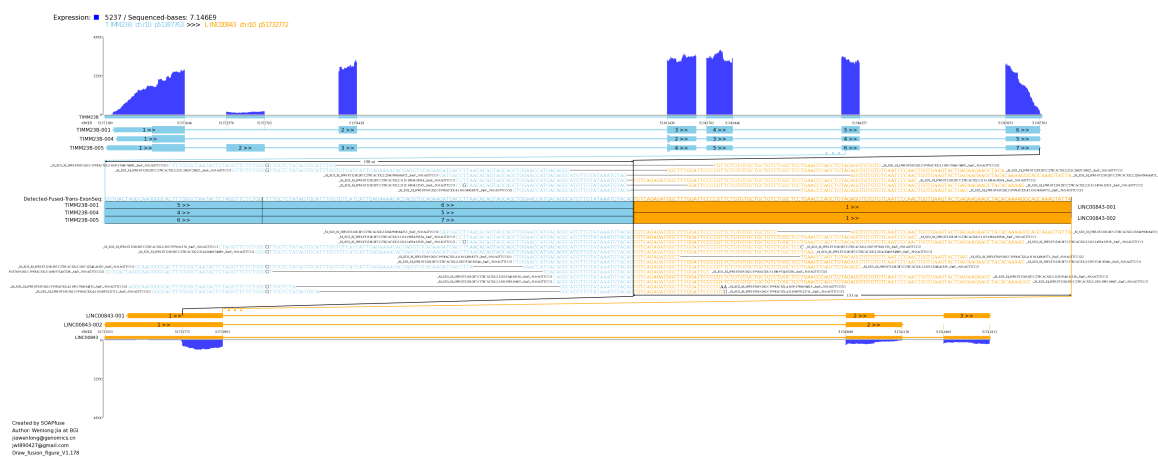

B

TIMM23B

LINC00843

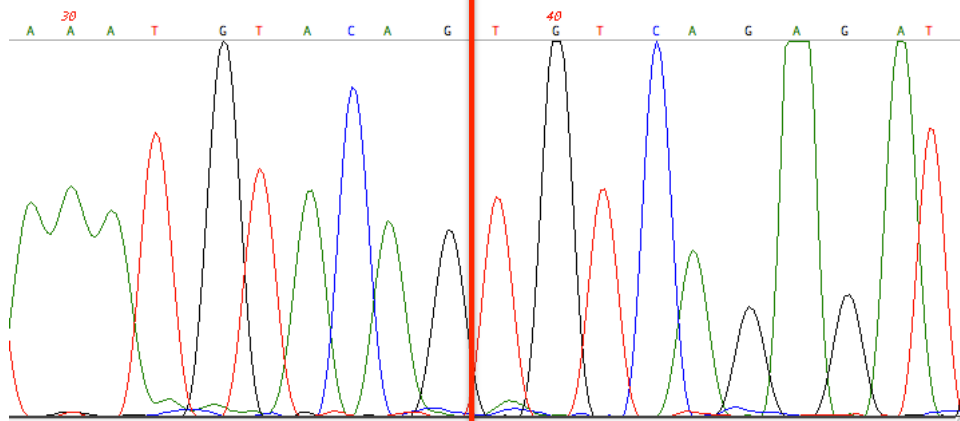

Fig. S4

Supplement: S4 Fig — A, structure of the fusion. B, validation of the fusion gene breakpoint using Sanger sequencing of RT-PCR product. (PDF) [file pgen.1005001.s004.pdf]

A

MLK4-FUT8

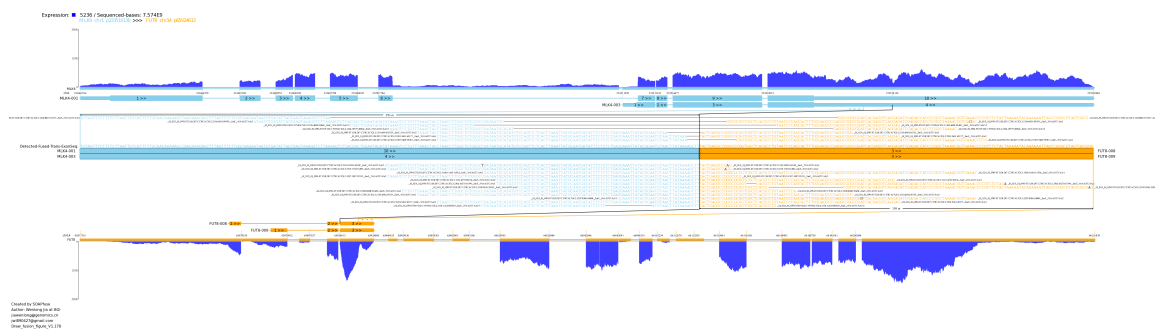

B

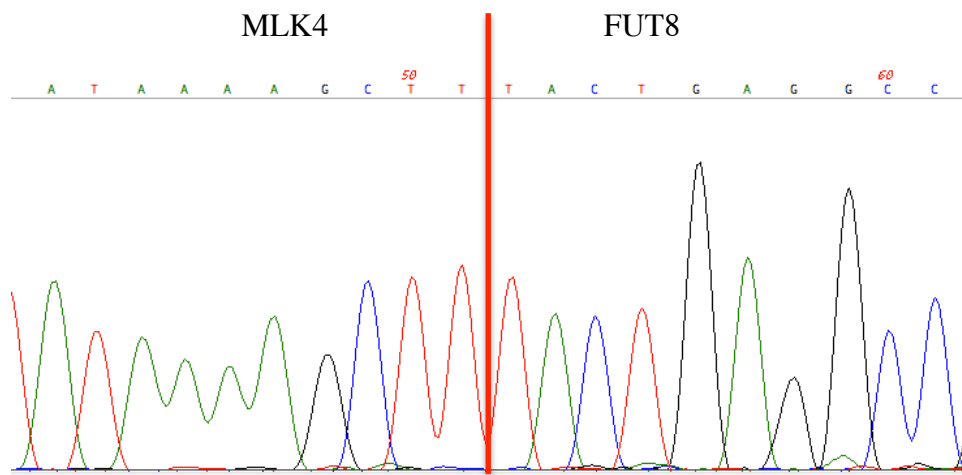

Fig. S5

Supplement: S5 Fig — A, structure of the fusion. B, validation of the fusion gene breakpoint using Sanger sequencing of RT-PCR product. (PDF) [file pgen.1005001.s005.pdf]

A

## TFDP1-GRK1

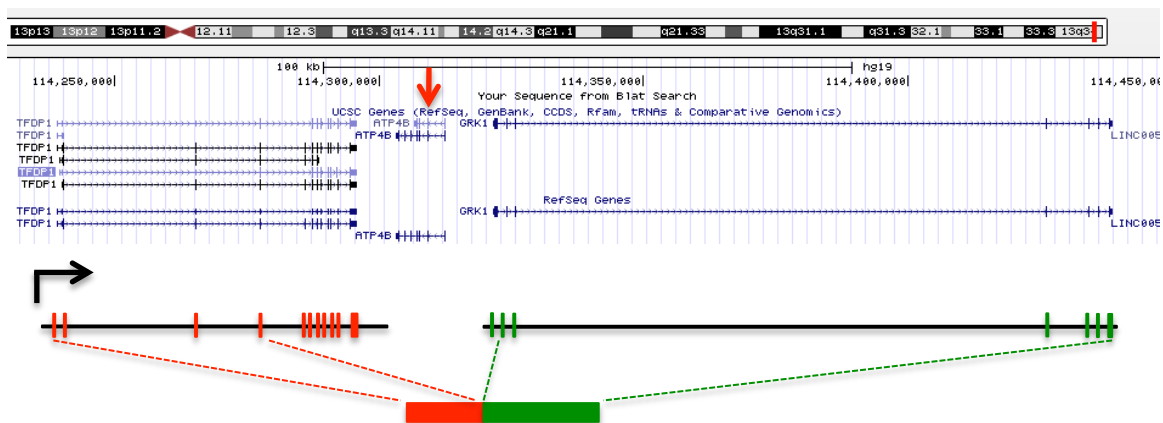

B

## VAMP1-CD27-AS1

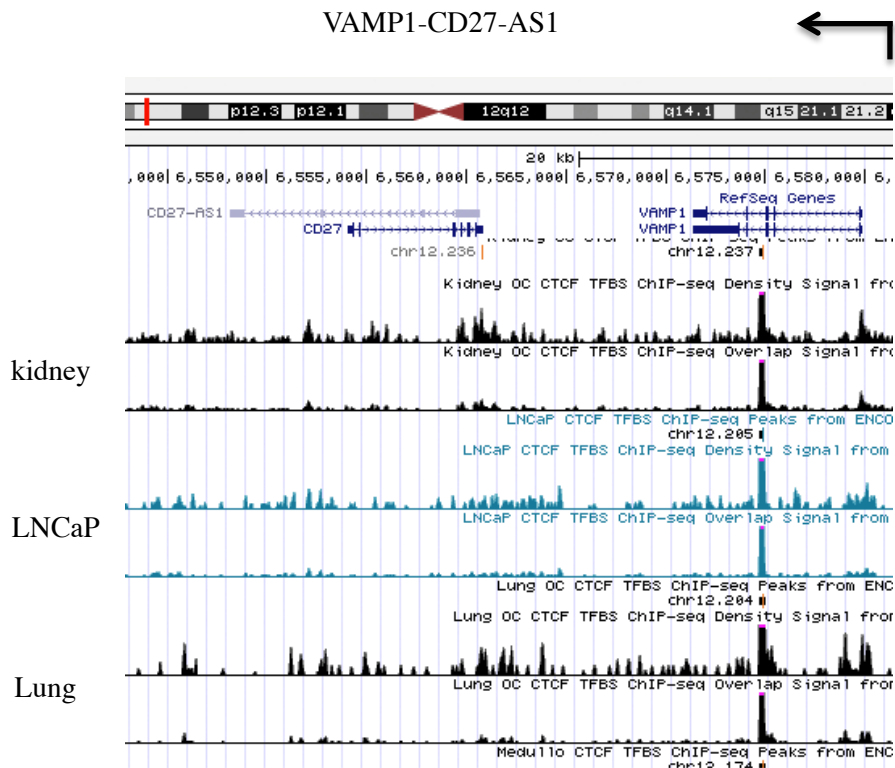

Fig. S6

Supplement: S6 Fig — A, TFDP1 and GRK1 are not immediate neighboring genes, as there is another gene, ATF4B, between the two. B, no obvious CTCF binding in-between VAMP1 and CD27-AS1. (PDF) [file pgen.1005001.s006.pdf]

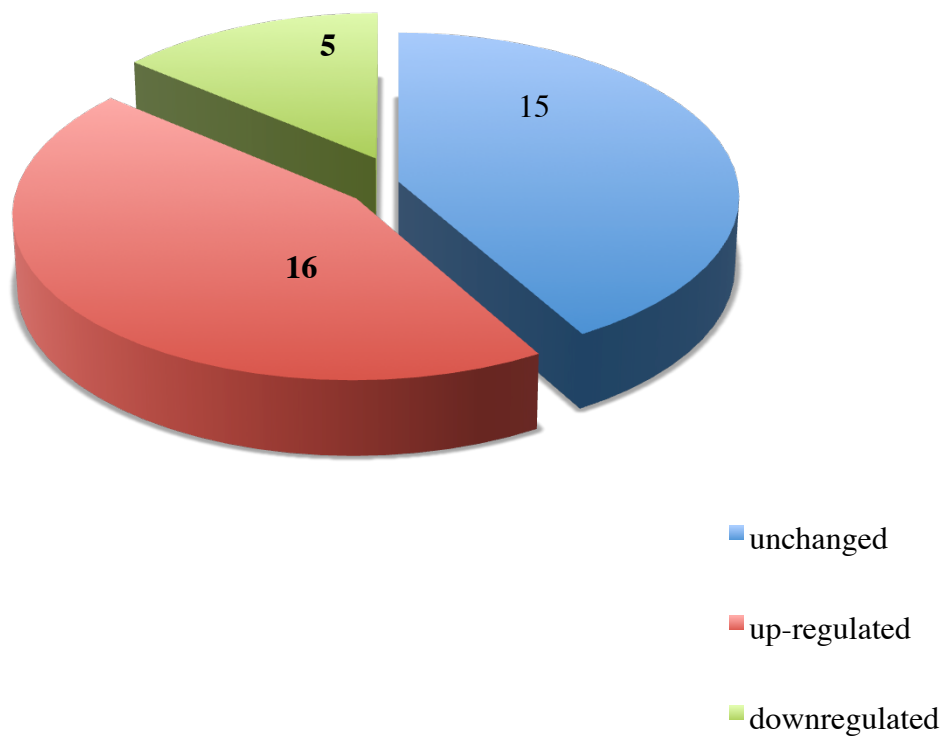

Fig. S7

Supplement: S7 Fig — (PDF) [file pgen.1005001.s007.pdf]

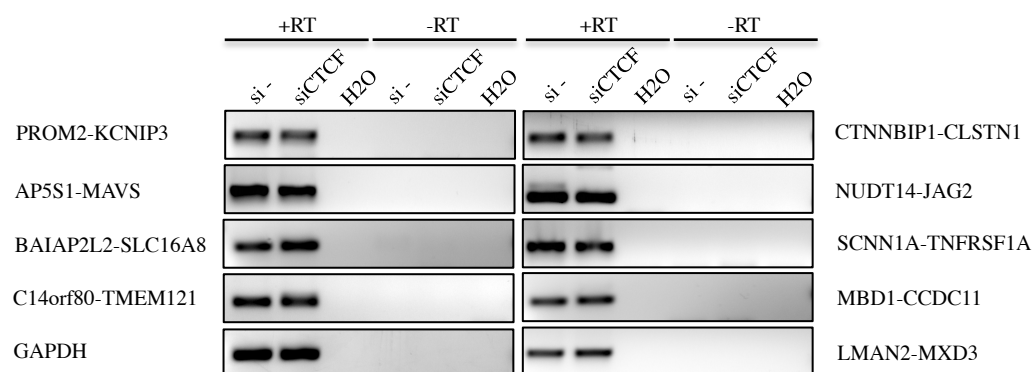

Fig. S8

Supplement: S8 Fig — Shown are 9 examples. (PDF) [file pgen.1005001.s008.pdf]

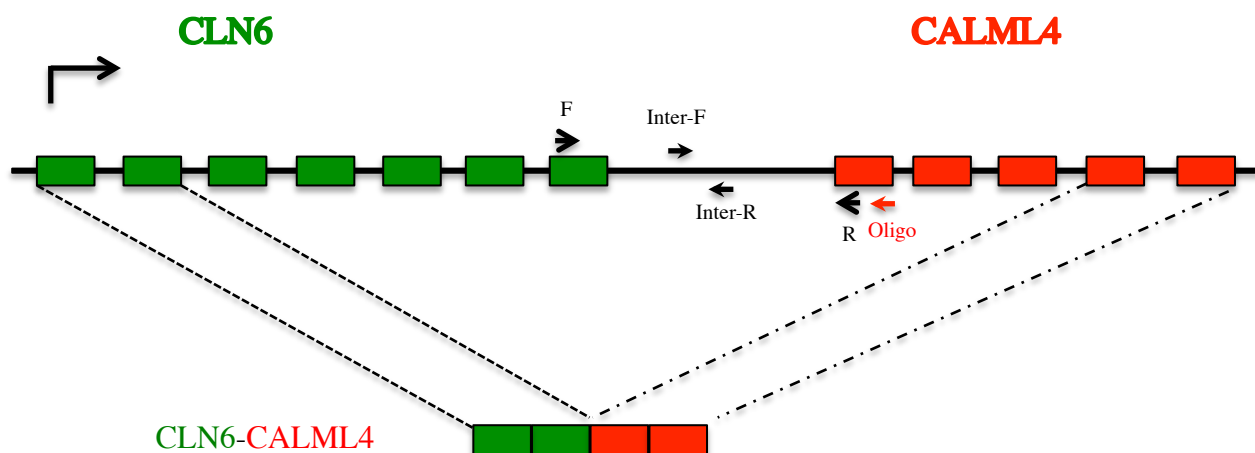

Fig. S9

Supplement: S9 Fig — Red oligo is the RT primer. F and R are the primer pairs for the long-range PCR. Inter-F and Inter-R are the primer pairs for data presented in Fig. 3D. (PDF) [file pgen.1005001.s009.pdf]

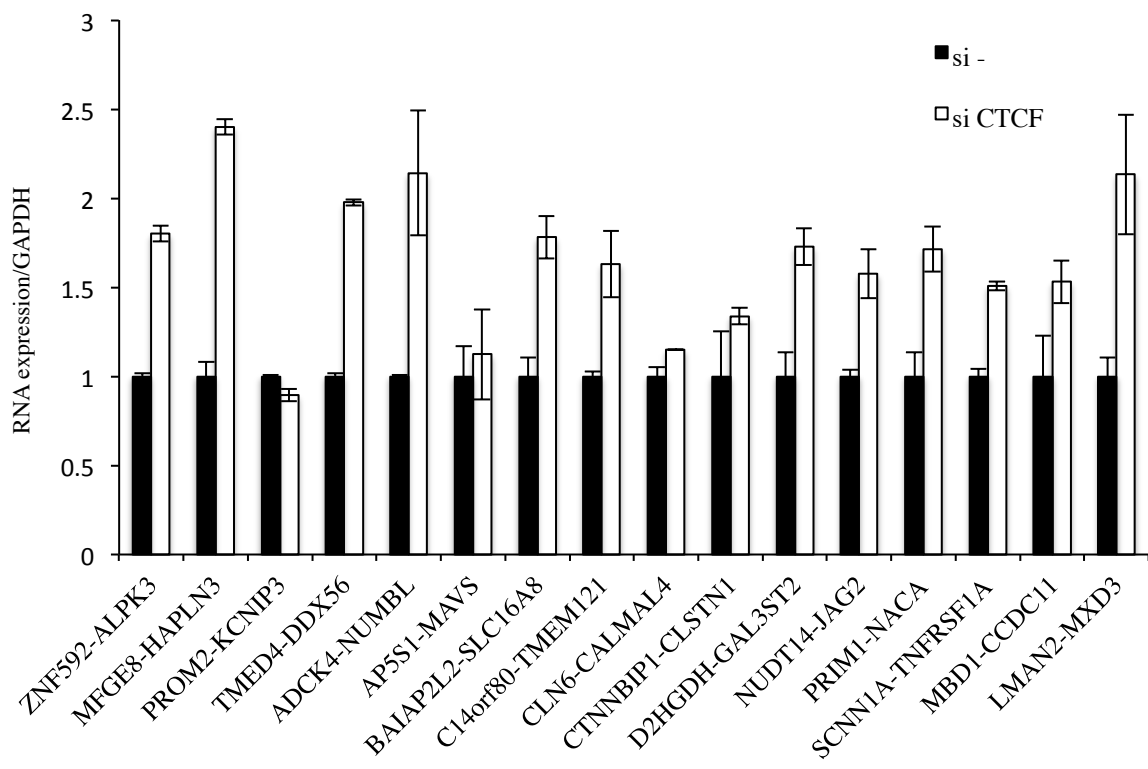

Fig. S10

Supplement: S10 Fig — (PDF) [file pgen.1005001.s010.pdf]

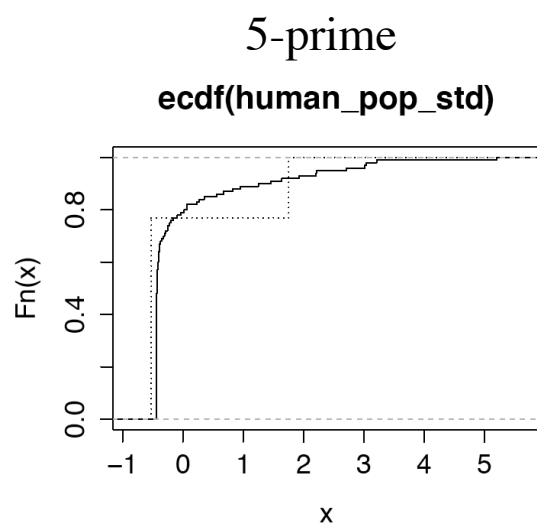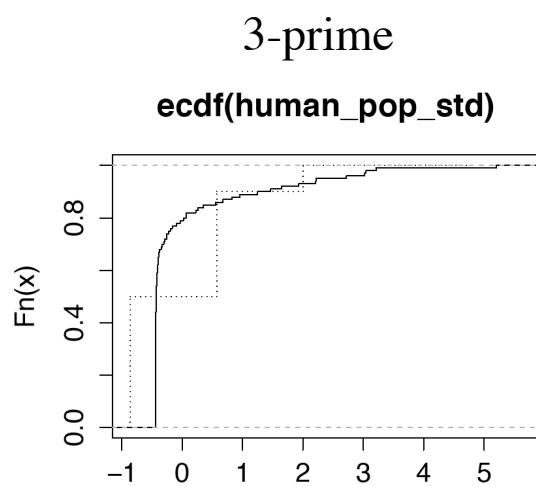

Fig. S11

Supplement: S11 Fig — (5 prime: D = 0.7692, p-value = 2.445e-06; 3 prime:D = 0.5, p-value = 0.02123) (PDF) [file pgen.1005001.s011.pdf]

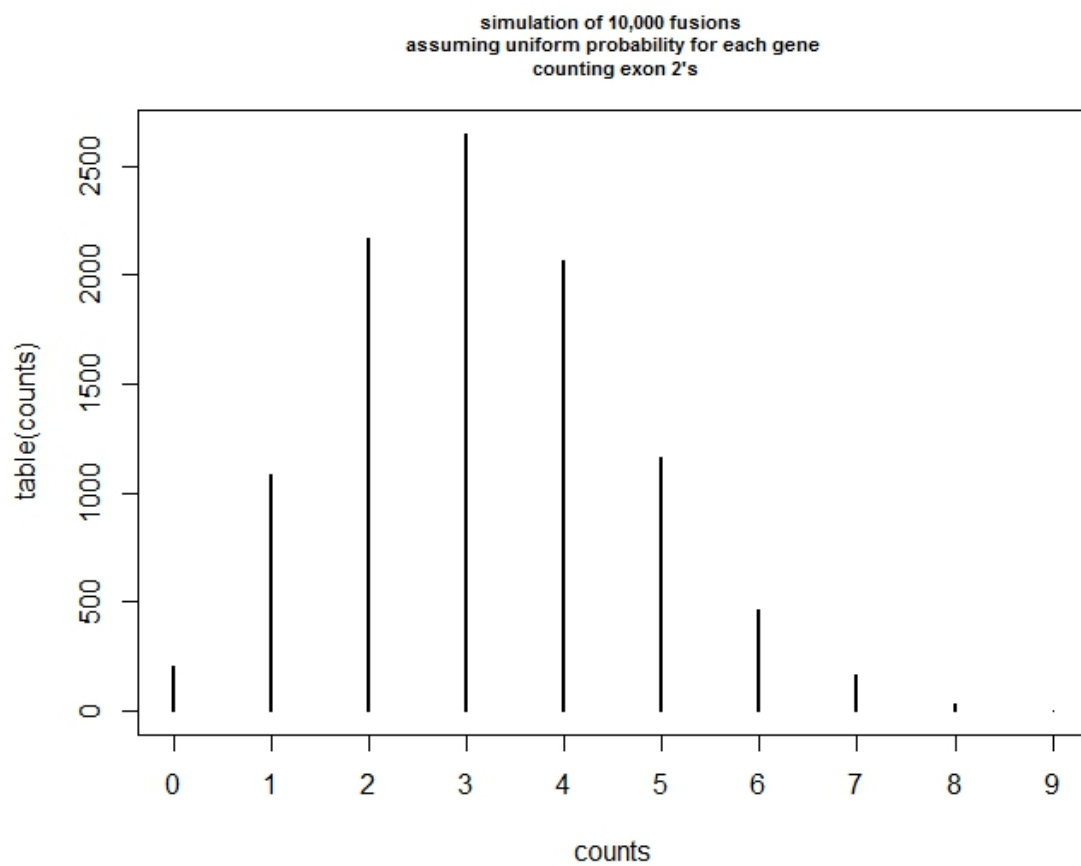

Fig. S12

Supplement: S12 Fig — 10,000 random sets of events with variation of the exon usage of the 16 3′ genes exons were simulated. The total number of fusions using exon2 in each set was counted and the results plotted. The peak at 3 indicates that out of 16 genes, the situation that having 3 genes using exon2 is more likely than any other number of genes using exon2. The fact that we have 11 genes using exon2 (falls outside the chart) is highly significant statistically (p = 1.718378e-05). (PDF) [file pgen.1005001.s012.pdf]
