# Supplementary material for: Discovery of CTCF-Sensitive Cis-Spliced Fusion RNAs between Adjacent Genes in Human Prostate Cells
Source: PLoS Genet. 2015 Feb 6;11(2):e1005001. doi: 10.1371/journal.pgen.1005001 (PMC4450057; doi:10.1371/journal.pgen.1005001)
Supplement: S2 Fig — Majority of the fusions are intrachromosomal. (PDF) [file pgen.1005001.s002.pdf]

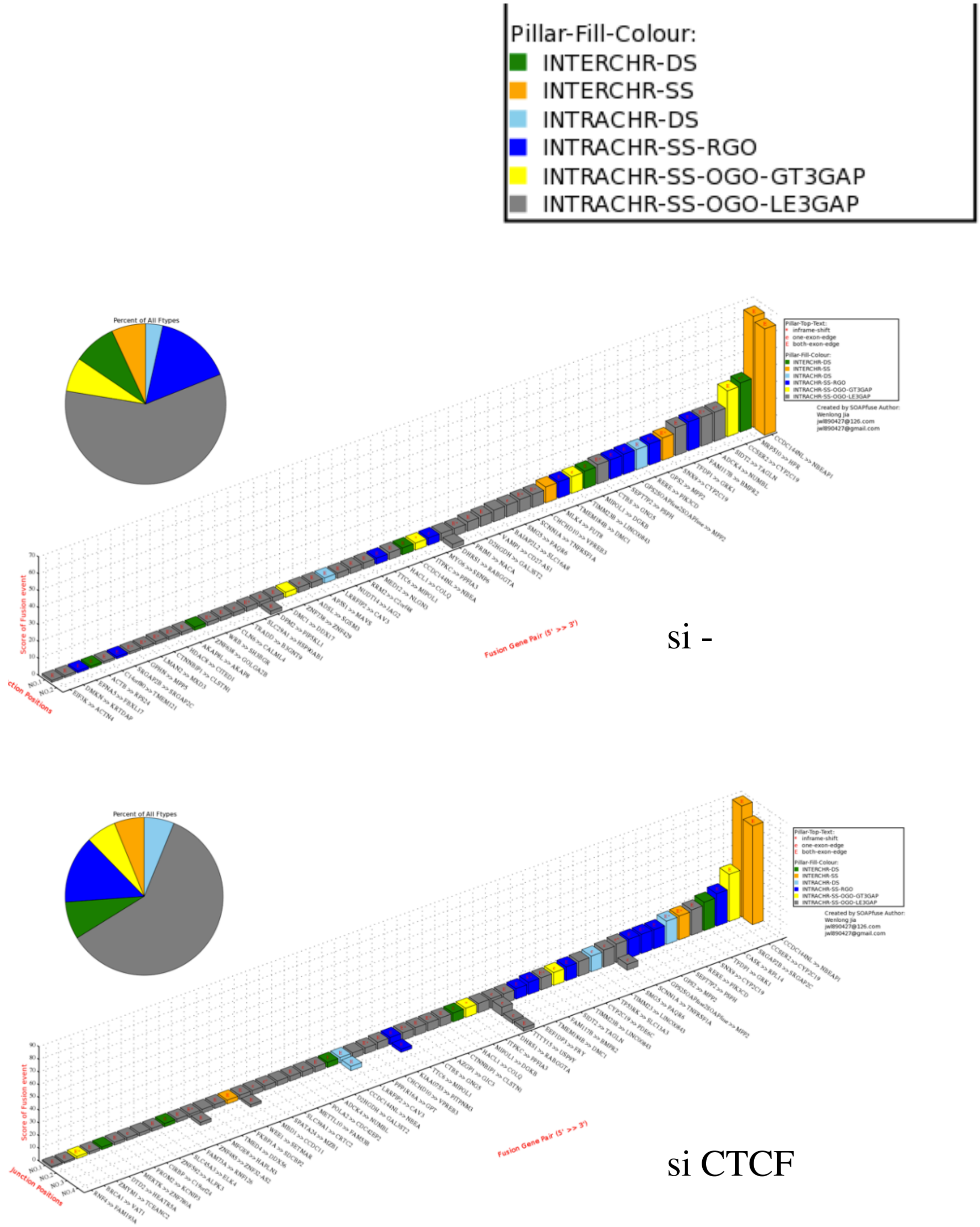
Pillar-Fill-Colour:
INTERCHR-DS
INTERCHR-SS
INTRACHR-DS
INTRACHR-SS-RGO
INTRACHR-SS-OGO-GT3GAP
INTRACHR-SS-OGO-LE3GAP
si -
si CTCF

Fig. S2
