# Supplementary material for: Discovery of CTCF-Sensitive Cis-Spliced Fusion RNAs between Adjacent Genes in Human Prostate Cells
Source: PLoS Genet. 2015 Feb 6;11(2):e1005001. doi: 10.1371/journal.pgen.1005001 (PMC4450057; doi:10.1371/journal.pgen.1005001)
Supplement: S1 Table — (PDF) [file pgen.1005001.s013.pdf]

Table S1. Summary of data from RNA-seq

experiment	sample	Lane	read length (bp)	No. of reads	Total read bases
hudsonalpha	si -	Lane1	50	76,717,578	7,460,951,100
		Lane2	50	72,501,444	
	si CTCF	Lane1	50	70,525,648	6,682,500,200
		Lane2	50	63,124,356	
axeq	si -	Lane1	101	33,535,145	6,774,099,290
		Lane2	101	33,535,145	
	Si CTCF	Lane1	101	31,420,003	6,346,840,606
		Lane2	101	31,420,003	
