# Supplementary material for: Discovery of CTCF-Sensitive Cis-Spliced Fusion RNAs between Adjacent Genes in Human Prostate Cells
Source: PLoS Genet. 2015 Feb 6;11(2):e1005001. doi: 10.1371/journal.pgen.1005001 (PMC4450057; doi:10.1371/journal.pgen.1005001)
Supplement: S2 Table — (PDF) [file pgen.1005001.s014.pdf]

Table S2. Full list of fusions in si- and siCTCF identified by RNA-seq and SOAPfuse analysis.

yellow shading stands for the presence of fusions in both samples

si-

up_gene	dw_gene	Fusion_Type
ACTB	RPS24	INTERCHR
ADCK4	NUMBL	INTRACHR-SS-0GAP
ADSL	SGSM3	INTRACHR-SS-0GAP
AKAP8L	AKAP8	INTRACHR-SS-0GAP
AP5S1	MAVS	INTRACHR-SS-0GAP
BAIAP2L2	SLC16A8	INTRACHR-SS-0GAP
C14orf80	TMEM121	INTRACHR-SS-0GAP
CCDC144NL	NBEA	INTERCHR
CCDC144NL	NBEAP1	INTERCHR
CCDC144NL	NBEAP1	INTERCHR
CCSER2	CYP2C19	INTRACHR-OTHER
CHCHD10	VPREB3	INTRACHR-SS-0GAP
CLN6	CALML4	INTRACHR-SS-0GAP
CTBS	GNG5	INTRACHR-SS-0GAP
CTNNBIP1	CLSTN1	INTRACHR-SS-0GAP
D2HGDH	GAL3ST2	INTRACHR-SS-0GAP
DHRS1	RABGGTA	INTRACHR-SS-0GAP
DHRS1	RABGGTA	INTRACHR-SS-0GAP
DMC1	DDX17	INTRACHR-SS-0GAP
DMKN	KRTDAP	INTRACHR-SS-0GAP
DPM2	PIP5KL1	INTRACHR-SS-0GAP
DPM2	PIP5KL1	INTRACHR-SS-0GAP
EFNA5	FBXL17	INTRACHR-OTHER
EIF3K	ACTN4	INTRACHR-SS-0GAP
FAM117B	BMPR2	INTRACHR-OTHER
GPHN	MPP5	INTRACHR-OTHER
GPS2	MPP2	INTRACHR-OTHER
GPS2SOAPfuse2SOAPfuse	MPP2	INTRACHR-OTHER

HACL1	COLQ	INTRACHR-OTHER
HDAC8	CITED1	INTRACHR-SS-0GAP
ITPKC	PPFIA3	INTRACHR-OTHER
LMAN2	MXD3	INTRACHR-SS-0GAP
LRRFIP2	CAV3	INTRACHR-OTHER
MED12	NLGN3	INTRACHR-SS-0GAP
MIPOL1	DGKB	INTERCHR
MLK4	FUT8	INTERCHR
MRPS10	HPR	INTERCHR
MYO6	SENP6	INTRACHR-OTHER
NUDT14	JAG2	INTRACHR-SS-0GAP
PRIM1	NACA	INTRACHR-SS-0GAP
RERE	PIK3CD	INTRACHR-OTHER
RRM2	C2orf48	INTRACHR-SS-0GAP
SCNN1A	TNFRSF1A	INTRACHR-SS-0GAP
SEPT7P2	PSPH	INTRACHR-OTHER
SIDT2	TAGLN	INTRACHR-SS-0GAP
SLC29A1	HSP90AB1	INTRACHR-SS-0GAP
SMG5	PAQR6	INTRACHR-SS-0GAP
SNX9	CYP2C19	INTERCHR
SRGAP2B	SRGAP2C	INTRACHR-OTHER
TFDP1	GRK1	INTRACHR-SS-0GAP
TIMM23B	LINC00843	INTRACHR-OTHER
TMEM184B	DMC1	INTRACHR-OTHER
TRADD	B3GNT9	INTRACHR-SS-0GAP
TTC6	MIPOL1	INTRACHR-OTHER
VAMP1	CD27-AS1	INTRACHR-SS-0GAP
WRB	SH3BGR	INTRACHR-SS-0GAP
ZNF638	GOLGA2B	INTERCHR
ZNF738	ZNF429	INTRACHR-OTHER
siCTCF		
ADCK4	NUMBL	INTRACHR-SS-0GAP
AZGP1	GJC3	INTRACHR-SS-0GAP
BRCA1	VAT1	INTRACHR-SS-0GAP

CASK	RPL14	INTERCHR
CCDC144NL	NBEA	INTERCHR
CCDC144NL	NBEAP1	INTERCHR
CCDC144NL	NBEAP1	INTERCHR
CCSER2	CYP2C19	INTRACHR-OTHER
CHCHD10	VPREB3	INTRACHR-SS-0GAP
CIRBP	C19orf24	INTRACHR-SS-0GAP
CTBS	GNG5	INTRACHR-SS-0GAP
CTNNBIP1	CLSTN1	INTRACHR-SS-0GAP
CYP2C19	PDE6C	INTRACHR-OTHER
D2HGDH	GAL3ST2	INTRACHR-SS-0GAP
DHRS1	RABGGTA	INTRACHR-SS-0GAP
DTD2	HEATR5A	INTRACHR-SS-0GAP
EEF1DP3	FRY	INTRACHR-OTHER
FAM117B	BMPR2	INTRACHR-OTHER
FAM73A	RNF126	INTERCHR
FKBP1A	SDCBP2	INTRACHR-OTHER
GPS2	MPP2	INTRACHR-OTHER
GPS2SOAPfuse2SOAPfuse	MPP2	INTRACHR-OTHER
HACL1	COLQ	INTRACHR-OTHER
ITPKC	PPFIA3	INTRACHR-OTHER
KIAA0753	PITPNM3	INTRACHR-SS-0GAP
LRRFIP2	CAV3	INTRACHR-OTHER
LRRFIP2	CAV3	INTRACHR-OTHER
MBD1	CCDC11	INTRACHR-SS-0GAP
MBD1	CCDC11	INTRACHR-SS-0GAP
MERTK	ZNF780A	INTERCHR
METTL10	FAM53B	INTRACHR-SS-0GAP
MFGE8	HAPLN3	INTRACHR-SS-0GAP
MFGE8	HAPLN3	INTRACHR-SS-0GAP
MIPOL1	DGKB	INTERCHR
POLA2	CDC42EP2	INTRACHR-SS-0GAP
PPP1R16A	GPT	INTRACHR-SS-0GAP
PROM2	KCNIP3	INTRACHR-SS-0GAP

RERE	PIK3CD	INTRACHR-OTHER
RNF4	FAM193A	INTRACHR-SS-0GAP
SCNN1A	TNFRSF1A	INTRACHR-SS-0GAP
SCNN1A	TNFRSF1A	INTRACHR-SS-0GAP
SEPT7P2	PSPH	INTRACHR-OTHER
SIDT2	TAGLN	INTRACHR-SS-0GAP
SLC39A1	CRTC2	INTRACHR-SS-0GAP
SLC45A3	ELK4	INTRACHR-SS-0GAP
SMG5	PAQR6	INTRACHR-SS-0GAP
SNX9	CYP2C19	INTERCHR
SPATA24	MZB1	INTRACHR-OTHER
SRGAP2B	SRGAP2C	INTRACHR-OTHER
TFDP1	GRK1	INTRACHR-SS-0GAP
TIMM23	LINC00843	INTRACHR-OTHER
TIMM23B	LINC00843	INTRACHR-OTHER
TMED4	DDX56	INTRACHR-SS-0GAP
TMEM184B	DMC1	INTRACHR-OTHER
TP53RK	SLC13A3	INTRACHR-SS-0GAP
TTC6	MIPOL1	INTRACHR-OTHER
TTC6	MIPOL1	INTRACHR-OTHER
TTTY15	USP9Y	INTRACHR-SS-0GAP
TTTY15	USP9Y	INTRACHR-SS-0GAP
TTTY15	USP9Y	INTRACHR-SS-0GAP
TTTY15	USP9Y	INTRACHR-SS-0GAP
WEE1	SETMAR	INTERCHR
ZMYM1	TCEANC2	INTRACHR-OTHER
ZNF485	ZNF32-AS2	INTRACHR-OTHER
ZNF592	ALPK3	INTRACHR-SS-0GAP
