# Supplementary material for: Discovery of CTCF-Sensitive Cis-Spliced Fusion RNAs between Adjacent Genes in Human Prostate Cells
Source: PLoS Genet. 2015 Feb 6;11(2):e1005001. doi: 10.1371/journal.pgen.1005001 (PMC4450057; doi:10.1371/journal.pgen.1005001)
Supplement: S3 Table — (PDF) [file pgen.1005001.s015.pdf]

Table S3. List of PCR and real-time PCR primers.

	Chimeric RNA	Forward primer (5'-3')	Reverse primer (5'-3')
INTRACHR-SS-0GAP			
1	SLC39A1-CRTC2	GTCCTGGTGATGGAGCAGAT	TGTGTATGCCAGTCGCAGTT
2	SMG5-PAQR6	GCTCCTTGAAGCTGCTCAGT	GGACCTGGTGGACTTGAAGA
3	METTL10-FAM53B	AGTTCAGCTTTGGAGGCAGA	TGGCCAAATGTGGTCAACT
4	TFDP1-GRK1	GAAGCCAACGGAGAACTCAA	GAATCTTCTTCTCCACCATAGCAC
5	ZNF592-ALPK3	CAGAGCCACATACCTCAGCA	CTGAGCAATGATGGAGCAGA
6	MFGE8-HAPLN3	AGCTACGGTAACGATCAGTGG	GTTGGCGCTGTTGGAGTAGT
7	KIAA0753-PITPNM3	TGAGCAGTACCTTCGGATCAT	TTCAGCCATCTCCTCTCTGG
8	CIRBP-C19orf24	ACCTGGAACAAGAGCTGGAG	CTGGCTCAGCATCATCTCA
9	PROM2-KCNIP3	AGGAGACTCAGCTCTTCCACA	GTGACAGGAGAGAAGGCACA
10	TP53RK-SLC13A3	TCCGTGCTGTTCTCGTGATA	TTGGACCTTAGTGCCACTTC
11	LINC00680-GUSBP4	AGCGAAGTGACAGGCTCAGA	TGGTGTGAGCAATCACCAT
12	TMED4-DDX56	GAGGCCAAGAAGCTGGTGTA	AAGTGGTGCCATGTGAGAGC
13	PPP1R16A-GPT	CCGACCTGAACGCAAAGT	CCTGTGGACAGGAAGACGTT
14	TTTY15-USP9Y	TTGACGTATGGAGCCAAGAA	GCTGTCGTTCCCTCCTACTG
15	ADCK4-NUMBL	TGTGTCCTGCAGAAGTCCAG	GGCTCTGCCGTAACTTGTTC
16	ADSL-SGSM3	CCAGGTTGATGCCTACTTCAG	TAGAGCTGGAAGGCTCCTCA
17	AKAP8L-AKAP8	CCAGAAGCATCTGAAGACCA	TAGCTGTAGGTTGCGCCTGT
18	AP5S1-MAVS	TCTTCGGTGCTGAGAAGTCA	TGCTCAGATTCTGGAGAGAGG
19	BAIAP2L2-SLC16A8	AACAGCAGCAGATCCAAGAA	CCTCTGAAGGACAACTGCTG
20	C14orf80-TMEM121	GCGCTCTGAGCTGGTCTTCT	ATCACCAGCGTGGTCAGG
21	CHCHD10-VPREB3	CTGGACTGTTCCACCACTCA	GCAGGAGAGTTGAGCCACTT
22	CLN6-CALML4	TTCCACCTCGACCTCTGGTT	CCACCATCAACATGGCTAGA
23	CTBS-GNG5	CTTGTAGTGACGCTGCAGGA	GAAGATACTCCAGTCAGCAGAGG
24	CTNNBIP1-CLSTN1	ATTCAGCAGAAGGTCCGAGT	TGTGACTATGCCGTGGTAGG
25	D2HGDH-GAL3ST2	ACGACATCGTGACTGACCTG	CTCTAAGTCCGAGTGCAGGAA
26	DHRS1-RABGGTA	GCAGACAGAACTGCTGAAGG	GAGGAGAGAAGTGTCAATCACG
27	DMC1-DDX17	GAGAGCTCAGAATTGCCAAGA	TCATATGGTGTCAGCCTTGC

28	DMKN-KRTDAP	TCACCTTCTTCCTCGGCTTC	CCAGTTCAGGAACTCATCAGC
29	DPM2-PIP5KL1	CTCCTGCTGCTCCTGTTTGT	TCTTGGAGGTGCTGAGGAAC
30	EIF3K-ACTN4	AACATGGACCTCTTGGAAGG	CAATGTTCTCGATCTGTGTGC
31	HDAC8-CITED1	GGTCCTGATTATGTGCTGGAA	TTCAGAGCCTTGGCAGAAGT
32	LMAN2-MXD3	AAGCTGTTCCAGCTGATGGT	CTTCTTCCTCCTGTGGATGG
33	MED12-NLGN3	CCGGCAACTTCAACAACAG	GGCCACACTGACTTGAACCT
34	NUDT14-JAG2	AGGAAGTGGCTTGCAAGGAG	GATCAGCAGCTCCTCATTCG
35	PRIM1-NACA	GAGGAGAATGAAGCTGAATCTG	TCCTGTTCTTCAAGCTCTGGT
36	RRM2-C2orf48	GACAGACTTATGCTGGAACTGG	TCAAGACGTAAGGCTGGTCA
37	SCNN1A-TNFRSF1A	GGAGTGGCCAAAGTCAACAT	GGTGAGGGACCAGTCCAATA
38	SIDT2-TAGLN	TCGCGCTCTTCTTCTTCTTC	GCCATAGGAAGGACCCTTGT
39	SLC29A1-HSP90AB1	TGTTGAGGTCAAGTCCAGCA	CTCTCCATGGTGCACTTCCT
40	TRADD-B3GNT9	GGGACCCTGAAACTCCACTT	CTTGCGTTGTTCTCCTCCTC
41	VAMP1-CD27-AS1	GTGCTGCCAAGCTAAAGAGG	GCTGAGGCAGGAGAATCACT
42	WRB-SH3BGR	GAAAGCTCGGACAGCTCAAT	TCCACCTCCTGTTGTCTTCA
43	AZGP1-GJC3	GGTGGAAGGAATGGAGGATT	TCATCTCCAACTCAGGCATCT
44	BRCA1-VAT1	AGCTGTGTGGTGCTTCTGTG	GTCACCTCTTCCTGCCACAT
45	DTD2-HEATR5A	GAGGACTGGTGATCTACGTGTG	CATTCCTTGCAGCAATCCTC
46	MBD1-CCDC11	GCAGTAGACTGGAGGCTTCTACA	CTTGCAGTCATTGTGCTGGT
47	POLA2-CDC42EP2	ATTCAGCCGAATACTCAAGCA	TGTCACCGAGGGTTACTTGTC
48	RNF4-FAM193A	AGCAAATTGGCCCTTTCCAT	GTGTAGCGCACTTCTGACCA
INTERCHR			
1	ACTB-RPS24	GGACTTCGAGCAAGAGATGG	TCCTCTGAAGTAGTCGGTTGG
2	CCDC144NL-NBEA	GGCTGGAGGACTAGAAGGAA	TGCTCCAGGACAGCTCTAGTT
3	CCDC144NL-NBEAP1	GGCTGGAGGACTAGAAGGAA	TTGCTCCAAGACAGGTCTAGTT
4	MIPOL1-DGKB	AACTTCAACATGCCAGAGAGG	CACAGGATTATTCCTTGCTTCG
5	MLK4-FUT8	GCCGATAATGCATATGTCCAG	TCTCGATCTCCTGACCTCGT
6	MRPS10-HPR	ACACATCAAGGAGCCAATCTG	GCCATTGTTCATTGATCAGC
7	SNX9-CYP2C19	TGATTCTCTCTCAGCCAGCA	TTCAGCAGGAGAAGGAGAGC
8	ZNF638-GOLGA2B	CAGCTTCCTCAGACGACACA	GCATGGTGGTCATTGCTCT
9	CASK-RPL14	TGTCCACTAAGCAATCAGAGAA	TACTCGCGGCGGTGATCTT

10	FAM73A-RNF126	GAGGTTGCAGTTAGCCGAGA	ACCACAGAAGAGGTCCACCA
11	MERTK-ZNF780A	CGAAGACTGCCTGGATGAAC	CTATTGCCTGAGAGCACAGC
12	WEE1-SETMAR	TCAGTATTGCTGTCCGCTTC	GCATTGTTGATGTTGCGAGT

INTRACHR-OTHER

1	CCSER2-CYP2C19	TTCGTCTCACCTTCCGTCCT	CCAACGACACGTTCAATCTC
2	GPHN-MPP5	TCGCCTCTCTACAGCTTCCT	CAGACCTAACTCTCGACATGGA
3	HACL-COLQ	GCAGAGCCTAGCAGACACAA	ACCACGCTTCTTCTGATCCA
4	ITPKC-PPFIA3	AGTTCCAGCCTCCAGACTCA	AGCCGCTTATTGTGGTCATC
5	TIMM23B-LINC00843	ACGAGGTGCAGAAGATGACC	AGACAGCAGCACACAGAACG
6	ZNF738-ZNF429	TGATGGAGTCTCGCTCTGTC	GCTGAGGCAGGAGAATCACT
7	EEF1DP3-FRY	ATAGCATGGTGCCAGACACA	GAACCGGAGGCTTGATGTAA
8	FKBP1A-SDCBP2	TACTAGGCAGAGCCGTGGAA	CTGAATGGCTTGGTCCACTT
9	SPATA24-MZB1	GCTCTCCAGTGTCAAGAGCA	TCCTCATCATCCAGTTGTGG
10	ZMYM1-TCEANC2	CGTTTCGCTTCGAAGATTGT	TCTGGCAAGAGAAGCCACTT
11	ZNF485-ZNF32-AS2	TGTGATGCTGGAGAACTATGG	TGAGGAAGCAGAGGAGACCT

INTERGENIC REGION OF 16 CIS-SAGE

	Chimeric RNA	FORWARD PRIMER (5'-3')	REVERSE PRIMER (5'-3')	STRAND_SPECIFIC_OLIGO_FOR_RT
1	ZNF592-ALPK3	GAGTGCAATGGCACAATCAT	ACCAGCCTGGACAATACAGC	AGACCAGGGAGCCAAAGAAC
2	MFGE8-HAPLN3	CCTTCCTTCCTTGGCTTCTC	CCAGCAGGTGGAATCTGAAC	CAAAGCGGTAAGGGAGGAAC
3	PROM2-KCNIP3	TCTGGTTGAGCAGAGGAAGC	TTGGTTCAAGTCACCTTGGAG	CCCAAAGGAAAGTGGAACAA
4	TMED4-DDX56	CCTCCACCTCCTGAGTTCAA	ACCTGCGGTCAGGAATTCAA	GCAACTGCCTGAGAGAGAGG
5	ADCK4-NUMBL	CCACACACACAGTGCCTCTT	TACGCCAATCCTGTGGGTTT	AAGAGATGGCCCAGGGGTCT
6	AP5S1-MAVS	TGCTGAGCTCCAGTTCCTGT	GGTCATTCTGTATCCTGCATTG	AGTGTTCCCGAAACTCTTGC
7	BAIAP2L2-SLC16A8	TCAGGAGATGTGAGGCTGGA	CCTGCCTGTTGTCCTCTTGT	GGGAAACGGAGGTAGGACTC
8	C14orf80-TMEM121	TGGAGGTCCTCACAACACTG	CAGCTGGCCTTAAGACAGGA	ACCCCAAAGTCACTGACAGG
9	CLN6-CALML4	GCAGCCTTCCCTCCTCTTAT	GCCTCTGTGTGGAAAGTGTG	GGATACAAGCTTCGGTGCTC
10	CTNNBIP1-CLSTN1	CAGCAGAGAGCTTGGAAACC	TTTCCTTTGCCTGCTGAAGT	AGATGGAGAGCCTCAACTCC
11	D2HGDH-GAL3ST2	AGCTCTGCTACTGGGCACAT	ACGGGCAGAAGCTTGAAA	CGAAGCCAAGACAGGTGAAT
12	NUDT14-JAG2	CAGCAGGACATTCACAGGAC	ATCGCACTCTCGTGGTGTCA	AAATAAGGCAGCGGGAGAG
13	PRIM1-NACA	AATTCCTGCTGGGTGCAGT	GGTTCAAGTGGCTGGGATTA	GTCACAGGGAGAACGGTGTT

14	SCNN1A-TNFRSF1A	AAGCCCTAACAGCCGATTCT	GTTTCGTGGAGGAGGTGAGA	CAGCTATGGCCTCTCACTCC
15	MBD1-CCDC11	TTCTGCCATTCTGATGAACC	ACTCGGGACTTCCTCTTCCA	GTGCGGAGAGATGGAGAAAG
16	LMAN2-MXD3	GCTTGAGCACAAGACGGAGT	CACCGTGACAGCCAAGTAGA	CCCGAGTGGTCTCAGACTTT
20 RANDOMLY SELECTED NEIGBORING PAIRS				
1	AGBL2-MTCH2	CCACCCTGATTCTGCCTAAG	CAGGAAGCTGACACACTTGC	
2	GPR108-C3	AGGAGGACGAGGAGGATGTT	TCAGCACAGTCTTCTCACTGG	
3	APLP2-ST14	GATGCTGAGGAAGAGGCAGT	GTTGTTGACTGGCAGGAACTC	
4	BLVRB-SERTAD3	ACTGCAGGCTGTGACTGATG	GGCTTGCTGGTAGCTCTGAA	
5	C19orf18-ZNF256	AGTCCACGCACCTACTTCCA	GCATCACATCGTGGTACAGG	
6	DNAJB11-AHSG	CAGGAGCGAAGCTATGGAAG	GGTGGTTTCCAGGGTGTCTA	
7	FAM179B-PRPF39	CATCTATGCGGCTGCTACAA	TGCCTGTGCTGCCATTACTA	
8	KLK3-KLK2	CCAAGTTCATGCTGTGTGCT	GGGAATGCTTCTCACACTCC	
9	MUL1-CAMK2N1	CACAAGATGGTGTGGAATCG	GGTGCCTTGTCGGTCATATT	
10	PAK1IP1-TMEM14C	AGGCTGACGTGTCTTGGAGT	ACTGAGCCAGTGTCCTGCAT	
11	RNF213-ENDOV	TGCTAGAGCTGCACGAAATG	CTTTCACGAAGGACACGTCA	
12	SLC25A44-PMF1	CCTCCATCCTCACCAATCC	GCTGCAACTGGTAGAAGCACT	
13	SLC44A4-NEU1	ACACGCTCTTCCTCTGCTTC	ACAGCAGTTGCTCCATGGTC	
14	TMEM107-VAMP2	TCTTCATATTCGAGCGTTGG	GTCTGCTGCAGTCTCCTGTT	
15	TXNDC12-RAB3B	TTATGTCAGTGCCGAGCAAG	ATCAGCATAGCGGAAGAGGA	
16	UXT-ELK1	CAACAGCCTCACCAAGGACT	ACTGCCACAGCGTCACAGAT	
17	ZNF383-LOC101927667	TGGTTGGCAGAGAGCTTACA	GCTCCTAATGATCTTGAGAGGTG	
18	DERL2-DHX33	CCAATCAACCTGGTGGAATAAG	TCCTGGTGTCTTCTGAGGTG	
19	FHL3-SF3A3	CCCTACTGTGTGGCCTGTTT	GAGTGCGGTGATCAGAATTG	
20	DDX5-POLG2	CCCAAGTTGCTTCAGTTGGT	TCTGAGCAAGGCCATAAGGT	
Others	MALAT1	GACGGAGGTTGAGATGAAGC	ATTCGGGGCTCTGTAGTCCT	
	ADCK4	ACAGAGACTGTGTCCTGCAGAAGT	AGGATCATCACTGCCTCCAC	
	NUMBL	ACCGCAGCAGCAGCAGTC	GGCTCTGCCGTAACTTGTTC	
